# Clinical expert consensus document on bailout algorithms for complications in percutaneous coronary intervention from the Japanese Association of Cardiovascular Intervention and Therapeutics

**DOI:** 10.1007/s12928-024-01044-y

**Published:** 2024-12-03

**Authors:** Takayuki Ogawa, Kenichi Sakakura, Satoru Sumitsuji, Makoto Hyodo, Junichi Yamaguchi, Hiroaki Hirase, Takehiro Yamashita, Kazushige Kadota, Yoshio Kobayashi, Ken Kozuma

**Affiliations:** 1 Division of Cardiology, Department of Internal Medicine, The Jikei University School of Medicine, Tokyo, Japan; 2 Division of Cardiovascular Medicine, Saitama Medical Center, Jichi Medical University, Saitama, Japan; 3 Cardiovascular Medicine, Future Medicine, Osaka University, Osaka, Japan; 4 Department of Cardiology, Kokura Memorial Hospital, Kitakyushu, Japan; 5 Department of Cardiology, Tokyo Women’s Medical University, Tokyo, Japan; 6 Takaoka Minami Heartcenter, Takaoka, Japan; 7 Sapporo Kojinkai Memorial Hospital, Sapporo, Japan; 8 Department of Cardiovascular Medicine, Kurashiki Central Hospital, Kurashiki, Japan; 9 Department of Cardiovascular Medicine, Chiba Graduate School of Medicine, Chiba, Japan; 10 Department of Cardiology, Teikyo University Hospital, Tokyo, Japan

**Keywords:** Percutaneous coronary intervention, Complication, Bailout

## Abstract

The efficacy and safety of percutaneous coronary intervention (PCI) for coronary artery disease has been established, and approximately 250,000 PCI procedures are performed annually in Japan. However, various complications including life-threatening complications can occur during PCI. Although several bailout procedures have been proposed to address complications during PCI, it is critically important for operators to manage each complication in real catheter rooms with confidence even in emergent situations. Standard bailout methods including specific techniques should be clarified as algorithms and shared with inexperienced operators as well as experienced operators. The Task Force of the Japanese Society for Cardiovascular Intervention and Therapeutics (CVIT) has developed the expert consensus document on bailout algorithms for complications in PCI.

## Introduction

Approximately 250,000 percutaneous coronary interventions (PCIs) procedures are performed annually in Japan [1]. PCI is less invasive than coronary artery bypass grafting surgery, and can be applicable for patients with high surgical risk. Furthermore, improved long-term patency with drug-eluting stents has expanded the indications for PCI [2]. However, various complications can occur in PCI. Because some of these complications can develop to the life-threatening complication, it is important to estimate the risk before PCI procedures and prevent complications. It is also important to detect early sing of complications, which requires comprehensive understanding for patient’s situation including vital sings. In addition, operators should prepare appropriate devices for bailout in their catheter rooms and have the knowledge regarding how to use devices in bailout procedures. However, the prompt bailout procedure can be difficult even in experienced operators, because unpredicted complications may trigger a near-panic situation. Furthermore, the bailout procedure without considering patient’s vital sings may worsen the situation. It is important to share the situation of complications and the bailout procedures among all staff members in the catheter room. Several literatures regarding algorithms for complications have been published, but the comprehensive documents have not been published from Japan [3, 4]. This expert consensus document outlines the algorithm for each complication and aims to present the methods as a flowchart for all operators and staff members in PCI.

## Overview

**Fig. 1:**
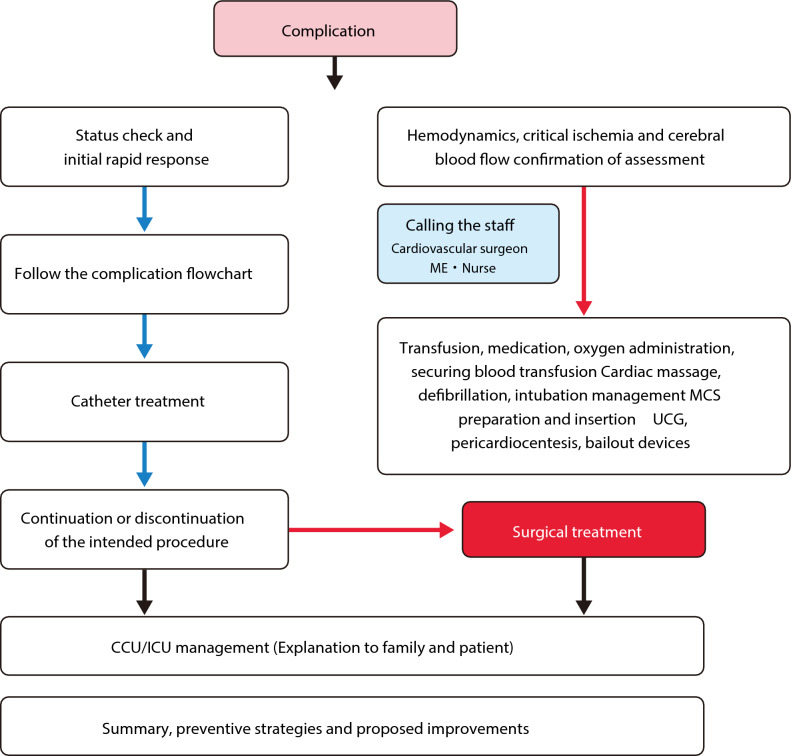
Flowchart for PCI complications. (Green right arrow) Successful (YES). (Red right arrow) Unsuccessful (NO). (Black right arrow) Progress

Figure 1 presents a general flowchart of the complication management strategy. When a complication occurs, it is important to diagnose the type of complications and evaluate the patient’s situation accurately. Feasible procedures should be implemented immediately after hemodynamic assessment. It is recommended to recruit additional staff members including cardiologists and cardiovascular surgeons. Not only bailout procedures but also hemodynamic support should be performed simultaneously to prevent cerebrovascular damage. All staff members in a catheter room should work well together for multiple tasks including preparation of bailout devices and mechanical support devices, explaining the situation to the patient’s family, and securing a bed in the Intensive care unit. It is recommended to have various bailout devices such as coils, covered stents, and several types of guided extension catheters (GEC) and microcatheters in the catheter room. All staff members in the catheter room should share how to use these devices and where these devices are located. Because the bailout procedure requires the multidisciplinary approach, it is recommended to establish protocols for various complications in each catheter room. It may be useful to train all staff members through simulation practice. We should not stick to percutaneous bailouts. We should not hesitate to ask surgeons for surgical bailout when the surgical bailout is better than the percutaneous bailout. Occasionally, minor complications, which are initially judged to be treated with percutaneous bailout, can become fatal complications. In such situations, it is important to consult surgeons immediately for surgical bailouts. It is necessary to build a good relationship with cardiovascular surgeons through regular conference, which foster a frank exchange of views in emergent situation. Even when the surgical bailout is chosen for complications, some catheter procedures are usually necessary, because open surgery requires time for preparation. Interventional cardiologists and cardiovascular surgeons should cooperate with each other as a heart team to achieve the most effective treatment for patients.

Occasionally, the experienced staff members including cardiovascular surgeons, non-invasive cardiologists, clinical engineers, radiologists, and nurses may recognize complications earlier than the operators and may provide additional perspectives on management. Furthermore, if time permits, it may be useful to consult more experienced doctors in other hospitals (or device company staff) by phone to compliment the bailout strategy of operators.

Once a serious complication occurs, a case conference should be held with all relevant staff members, including a third-party member, if possible, to review the causes and the procedures and to prevent future ones. Regardless of treatment success, team debriefing is recommended for cases with complications. Team debriefing can provide a forum for all staff members to discuss various issues. Efforts to learn from complications will strengthen the team and improve future procedural performance.

## Severe coronary artery dissection caused by catheters

[Mechanism]

Severe coronary artery dissection is defined as coronary artery dissection that reduces blood flow in the true lumen and causes fatal myocardial ischemia. Severe iatrogenic coronary artery dissection, regardless of whether it is caused by diagnostic catheters or guiding catheters, begins with the formation of dissection at the catheter tip. Such dissection can expand circumferentially and longitudinally and compress the true lumen, which results in the reduced coronary blood flow and subsequent myocardial ischemia. When dissection is caused by the catheter tip, especially when the catheter tip pressure is wedged, contrast injection makes dissection extend further [5].

Coronary artery dissection does not immediately become fatal when a guidewire is already placed into the distal true lumen, because operators can expand the true lumen via the guidewire and secure blood flow in the true lumen if necessary. However, when a guidewire is not placed into the distal true lumen, the expansion of coronary dissection can be fatal because of difficulty to secure the blood flow in the true lumen. Even when a guidewire is placed into the distal true lumen, coronary dissection can be fatal if operators cannot expand the true lumen because of severe stenosis or calcification.

[Incidence]

Catheter-induced severe coronary artery dissection occurs with similar frequency between the right coronary artery (RCA) and left coronary artery (LCA). Eshtehardi et al. reported that left main trunk (LMT) dissection occurred in 0.06% of diagnostic catheterizations and 0.1% of PCIs, with 42.1% of LMT dissections extending into the major branch and 2.6% of those extending into the ascending aorta [5, 6]. Two retrospective studies reported the incidence of iatrogenic aortic dissection as 0.006–0.01% with diagnostic catheters and as 0.098–0.12% during PCI [7, 8].

[Management]

**Fig. 2:**
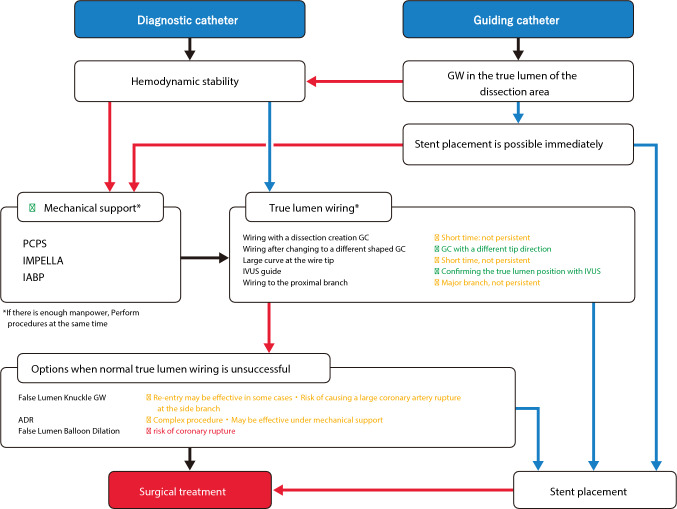
Flowchart for severe coronary dissection caused by catheter. (Green right arrow) Successful (YES). (Red right arrow) Unsuccessful (NO). (Black right arrow) Progress. (Green circle) Recommended. (Yellow circle) Not recommended, but no other method is available, and successes have been reported. (Red circle) Not recommended. GW, guidewire; PCPS, percutaneous cardiopulmonary support; GC, guiding catheter; IABP, intra-aortic balloon pumping; IVUS, intravascular ultrasound; ADR, antegrade dissection re-entry

Managing severe coronary artery dissection consists of “securing systemic circulation, especially cerebral blood flow” and “securing blood flow in the true lumen.” The management flowchart is shown in Fig. 2. It is ideal to perform the stent placement in the true lumen to secure the blood flow before circulatory collapse. In that case, further interventions are rarely required, and the clinical outcomes are mostly favorable. However, it is difficult to estimate the time from the onset of coronary artery dissection to hemodynamic collapse. It is also difficult to estimate the time required for operators to advance the guidewire into the distal true lumen. Moreover, it requires several staff members and additional time to prepare and insert mechanical support devices. Therefore, it is difficult to decide whether to prioritize securing blood flow in the true lumen or to start mechanical support devices. When a guide catheter is already engaged, it is reasonable to adjust the tip direction of guide catheter and advance the guidewire in a very short time. Note the guidewire would advance into the false lumen without adjustment of the tip of guide catheter. However, since it takes at least several minutes to exchange the guide catheter or to perform intravascular ultrasound (IVUS)-guided wiring, the decision to proceed with the above procedures or prioritize mechanical support device insertion should be based on the predicted success rate of the above procedures and the estimated time required for the above procedures. Furthermore, if severe coronary artery dissection is caused by diagnostic catheter, bailout procedures would begin with the exchange of arterial sheath, which would require additional time and effort. In such cases, mechanical support device insertion should be prioritized. For mechanical support insertion, sterilization of puncture site and insertion of femoral sheath should be performed in parallel with mechanical support system priming. If the number of staff members is sufficient, it may be reasonable to divide staff members into the two teams. One team prepares and manages mechanical support devices, whereas another team performs wiring procedure to secure blood flow in the true lumen.

The following steps should be considered to secure blood flow in the true lumen under mechanical support devices or when hemodynamics is maintained.

### Adjust the tip direction of guide catheter and select the different shape of guide catheter [Recommended]

In the case of dissection with guide catheter, the tip direction of guide catheter should be adjusted, and the guidewire should be advanced as described above. If the guidewire is advanced only into the false lumen after adjusting the tip direction of guide catheter, it is recommended to switch to the guide catheter with different tip shape. In the case of dissection with diagnostic catheter, a guide catheter with different shape from diagnostic catheter should be selected after exchanging the arterial sheath. When the dissection lumen is enlarged, it may be useful to select the different shape of guide catheter tip such as larger curve (e.g., JL 4) than initially selected curve (e.g., JL3.5). When the dissection lumen is enlarged, modifying the shape of the guidewire tip curve, such as making the guidewire tip curve larger than usual, may enable true lumen selection. However, these efforts do not guarantee the successful true lumen wiring. Despite all efforts, operators sometimes cannot advance the guidewire into the true lumen. When operators advance the guidewire into the true lumen, operators would feel less resistance as compared to the resistance when operators advance the guidewire into the false lumen. However, since operators tend to be biased optimistically in troubleshooting situations, operators may ignore the resistance in the false lumen, and may keep advancing the guidewire into the false lumen, which expands the false lumen. When operators feel minor resistance during the guidewire manipulation, it is necessary to carefully judge whether the guidewire is truly within the true lumen. It is important not to inject the contrast media, but to use IVUS for confirmation whether the guidewire is within the true lumen.

### IVUS guidance [Recommended]

When operators cannot advance the guidewire into the false lumen, the best option is IVUS-guided wiring. Operators should advance the IVUS catheter via the guidewire placed in the false lumen and recognize the starting point of the false lumen. Then, operators should confirm the position of the remaining true lumen at the starting point of the false lumen. Operators can recognize the position and direction of the remaining true lumen under fluoroscopy using information including the direction of side branch, the position of IVUS catheter within the proximal true lumen and guide catheter, and the relative positions of the guidewire and the IVUS catheter. Then, operators can perform efficient IVUS-guided wiring to secure true lumen quickly. Although optical coherence tomography (OCT)-guided PCI has recently increased, OCT is contraindicated in this setting. OCT imaging requires the blood clearance flush, which would cause the false lumen enlargement. Even if the OCT equipment is already placed on a sterile table, an IVUS catheter should be prepared in the case of severe coronary artery dissection.

### Major side-branch wiring

Major side branches should be secured if the dissection occurs close to major side branches. However, it is sometimes difficult to secure the side branch because of complex coronary anatomy, especially when the entry point of dissection locate at the proximal to the bifurcation of the major side branch. Securing the true lumen of the target vessel is the most important. Therefore, operators should not spend excessive time for side-branch wiring. If the guidewire in the main vessel is confirmed to be in the true lumen using IVUS, it is safe to use a double-lumen catheter for side-branch wiring.

### Contrast agent injection [contraindicated]

Injection of contrast agent is contraindicated in severe coronary artery dissection, because it expands the dissection lumen and makes the true lumen wiring more difficult. Operators should put all efforts to avoid contrast injection by using IVUS. Minimum injection of contrast agent should only be used when it is truly necessary. In particular, when the tip of guide catheter pressure is wedged, injection of contrast agent can cause the extension of dissection, possibly retrograde dissection to the ascending aorta; therefore, such injection is the absolute contraindication [5]. The following options should be considered when the conventional true lumen wiring is unsuccessful:

### False lumen knuckle guidewire

When operators cannot advance the guidewire into the true lumen despite all efforts, it is an option to advance a knuckle guidewire into the false lumen to communicate with the true lumen at the middle or distal part of coronary artery, which would create re-entry at the middle or distal part of coronary artery. Creating re-entry may decrease the pressure in the dissected false lumen, increase the true lumen blood flow, improve hemodynamics and symptoms, and potentially provide more time for operators to perform subsequent procedures. According to experiences of one member of this task force, a slippery intermediate guidewire may be preferable from the perspective of creating communication with the true lumen without damaging the adventitia, while most experiences with false lumen knuckle guidewire are derived from PCI for chronic total occlusion (CTO) rather than from severe coronary artery dissection. However, since this method does not allow operators to control the re-entry location, there is a risk of expanding the false lumen. Furthermore, if the knuckle guidewire migrates into a side branch, there is a risk of large coronary artery rupture. When operators try the knuckle guidewire in the false lumen, it is important to confirm that the guidewire advances into the main vessel, not side branch. Furthermore, it is also important not to push the knuckle guidewire forcefully.

### ADR: antegrade dissection re-entry

When operators cannot advance the guidewire into the true lumen despite all efforts, antegrade dissection re-entry (ADR) may be an option. In ADR, operators advance the guidewire from the false lumen to the true lumen using IVUS guidance or dedicated devices. However, ADR is complex, and the use of stiff guidewire for re-entry is associated with the greater risk of coronary perforation.

### Penetrating guidewire without IVUS or dedicated device [contraindicated]

The true lumen wiring by stiff guidewire used for CTO PCI is contraindicated unless IVUS guidance or use of dedicated device, because of the high risk of coronary perforation and the low risk of successful wiring.

### False lumen balloon dilation [contraindicated]

Balloon dilation in the false lumen would not make re-entry into the true lumen, but enlarge the false lumen, like balloon dilation in the subintimal space in CTO PCI. It is contraindicated to use excessively large size balloon or scoring balloon because of the greater risk of coronary artery rupture.

Open chest surgery should be selected if “the true lumen wiring” or “stent placement in the true lumen” is unsuccessful. When severe coronary artery dissection occurs, it is important to share information with the cardiac surgery team immediately. Multidisciplinary treatment including surgical treatment should be performed before patients have irreversible damage.

One member of this task force experienced a complication that the coronary artery dissection extended to the ascending aorta and aortic valve. When discussing this case with a cardiac surgeon, we learned that the closure of the dissection entry site is the most important point in aortic dissection surgery. In other words, when coronary artery dissection extends to the aorta, “the closure of the dissection lumen within the coronary artery is necessary,” which should be achieved through PCI rather than surgical treatment. The same member experienced both cases with “favorable outcomes after closing the dissection lumen with a covered stent” and “unfavorable outcomes after hesitating to place a covered stent.” When multidisciplinary treatment including cardiac surgery is performed for coronary artery dissection extending to the aorta, it is important to provide the best treatment option as the heart team after discussion regarding the closure of dissection lumen within the coronary artery with cardiac surgeons.

## Coronary artery perforation

[Mechanism]

In this document, coronary artery perforation is defined as guidewire perforation that is caused by guidewires advanced outside the vessel in the peripheral coronary artery. Guidewire perforation occurs when the vessel wall is subjected to a force sufficient to disrupt the structure of the vessel wall at the contact point between the tip of guidewire and the vessel wall.

[Incidence]

The incidence of guidewire perforation ranges from 0.5 to 1.0% [9, 10] in literatures. However, the incidence of guidewire perforation may increase due to the recent development of complex PCI procedures.

[Predisposing conditions]

The elements of guidewire that influence the magnitude of the tip force exerted on the vessel wall are “slipperiness” and “deformation resistance.” Since the tip force reduction due to friction resistance is less in the use of “slippery guidewire” or “guidewire with deformation resistance,” large force tends to be exerted at the tip of guidewire. Note guidewires with deformation resistance include both guidewires with “high tip load” and guidewires with “strong shaft support.” The force exerted on the vessel wall by the guidewire tip is also influenced by the angle between the guidewire tip and the vessel wall. When the guidewire tip contacts the vessel wall at the angle near perpendicular, stronger force is exerted on the vessel wall. Therefore, special attention is necessary in “tortuous coronary arteries.”

[Management]

**Fig. 3:**
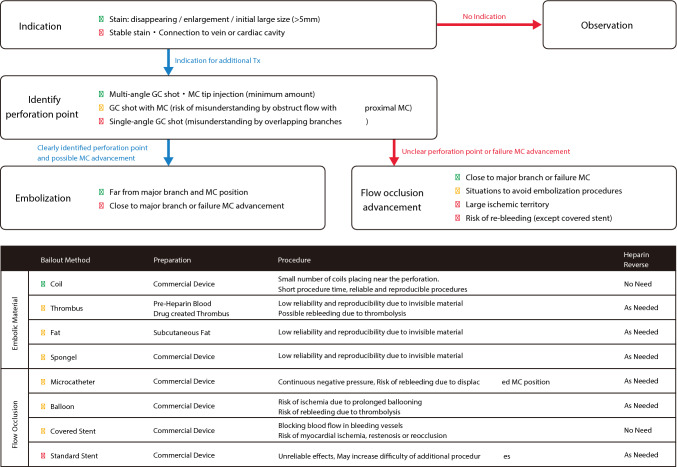
Flowchart for coronary perforation. MC, microcatheter

The management flowchart is shown in Fig. 3.

### Early detection

Early diagnosis is critically important. Unlike coronary artery rupture, there is a time lag between guidewire perforation and hemodynamic deterioration. Therefore, early detection of guidewire perforation can prevent serious complications such as cardiac tamponade. Causes that may prevent early detection are failure to confirm the position of guidewire tip and inadequate assessment of resistance when advancing the guidewire. Careful attention to the guidewire tip position, tactile resistance during the guidewire advancement, and the guidewire tip shape allow operators to consider the possibility of perforation and enable operators to detect perforation early through contrast injection from guide catheter or microcatheter.

### Identification of perforation site

Guidewire perforation can be confirmed by contrast injection from guide catheter or microcatheter. In the confirmation by contrast injection from guide catheter, it is important to observe the distal coronary artery sufficiently and to take a long time of cine-angiography to visualize the venous phase. Attention should be paid to the possibility of perforation occurring in the invisible areas on the fluoroscopy-monitor and the possibility of perforation-induced hematoma masked by the contrast in the veins. Careful observation should be routinely performed during the final contrast injection in PCI, even when guidewire perforation is not suspected. Operators should use adequate contrast volume for the final contrast injection to visualize the distal vessels sufficiently and continue cine-angiography until the end of venous phase to avoid missing the guidewire perforation.

When operator use a microcatheter to identify the perforation site, operators should advance the microcatheter near the suspected area. A tip contrast injection via the microcatheter can reveal the presence and location of guidewire perforation. To avoid air embolism, the contrast agent should be dripped into the lumen of the microcatheter while the guidewire is removed. Small-volume contrast injection with low pressure should be administered via the microcatheter to minimize hematoma expansion.

### Cardiac tamponade risk assessment

At the time of detecting perforation, most guidewire perforations are “localized hematomas.” These hematomas exist within the coronary perivascular tissues (fibroblasts and fat cells) and do not communicate with the pericardial space. The key issue at this point is to estimate the risk of future cardiac tamponade. The main mechanism that localized hematoma communicates with the pericardial space is the rupture to the pericardial space due to increased intra-hematoma pressure. Since hematoma enlargement precedes the rupture to the pericardial space, the absence of “hematoma enlargement” indicates a low risk of cardiac tamponade. Although it is difficult to judge “hematoma enlargement,” the absence of changes in the shape and size of contrast-filled hematoma over a few minutes suggests a significantly low risk of cardiac tamponade. In this situation, careful observation is appropriate. Conversely, if the hematoma enlarges over a few minutes, there is a significant risk of cardiac tamponade. A large initial hematoma size (> 5 mm) also warrants careful observation and assessment, because a large initial hematoma may indicate the greater risk of hematoma expansion. Rarely, communication with the pericardial space may exist at the beginning of guidewire perforation. In this situation, the risk of cardiac tamponade is high, and immediate action is necessary.

In addition to typical cardiac tamponade, where blood accumulates in the pericardial space, hematomas can also expand into the myocardium, which results in the tamponade-like situation (so-called “dry tamponade”). Even when the hematoma is confined to the myocardium, the assessment of “progressive hematoma enlargement and initial hematoma size” can predict hemodynamic deterioration.

### Embolization and blood flow blockage

There are two main approaches for addressing the risk of cardiac tamponade. The first approach is the use of embolic agents to reduce hematoma inflow and decrease intra-hematoma pressure. The other approach is to block the hematoma inflow by using microcatheter, balloon, or covered stent. When performing an embolization, it is necessary to identify the perforation site correctly. Because it is difficult to accurately identify the perforation site in the distal coronary artery by contrast injection from guide catheter, tip injection via microcatheter is recommended. One method is to inject contrast media from guide catheter after the microcatheter is advanced distally. In this method, operators identify the perforation site by confirming the absence of contrast leakage. However, this method is not recommended because the microcatheter may block blood flow, even when the microcatheter tip is located far from the perforation site. If the contrast volume is too large in tip injection via microcatheter, the perforation site may not be correctly identified because of abundant backflow of the contrast agent. Tip injection should be performed with a “very small amount” of contrast media at “low pressure.”

**Fig. 4:**
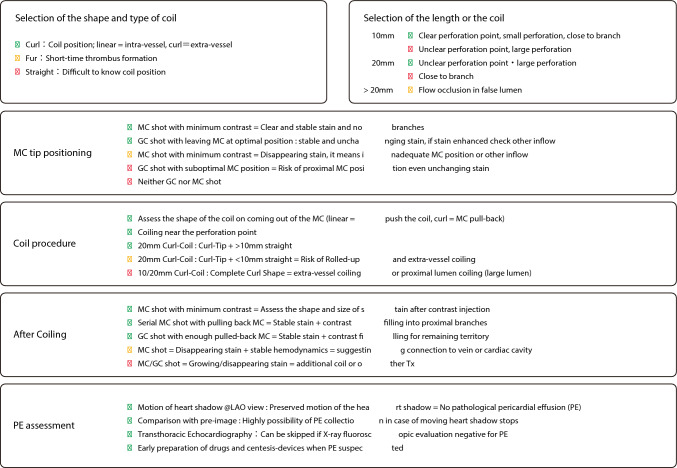
Coil placement techniques using curled coils

Various embolic agents such as coil, thrombi, fat, and gel foam have been widely used. Of those agents, coil is most used recently. Regardless of the agent used, embolization at the site distant from the perforation site requires a “larger amount” of embolic material because of the wider vessel lumen, which results in a larger ischemic area. It is important to place embolic agents close to the perforation site. Furthermore, curled coils are most effective, because these coils provide stronger blood flow obstruction. As guidewire perforation sites are often around distal coronary arteries with high flexibility, the curled shape can deform the vessel and more easily reduce intra-hematoma pressure. Additionally, curled coils assume straight shape within the vessel structure and curled shape outside the vessel structure, which allows operators to confirm the successful intravascular placement and to identify the perforation site at the transition between the straight and curved portions. The recommended coil placement techniques using curled coils are shown in Fig. 4.

When accurate identification of the perforation site is difficult, or when embolization devices cannot be used for any reason, blood flow obstruction using a microcatheter, balloon, or covered stent is an option. When using a microcatheter, negative pressure via microcatheter can make the distal vessel lumen collapse and block blood flow effectively. However, when a microcatheter or balloon is used to block blood flow, sufficient time is required to create a thrombus at the perforation site for the prevention of cardiac tamponade. Covered stent for jailing the branch with perforation ensures definitive blood flow blockage, but carries the risks of expanded ischemic area, chronic restenosis, and occlusion of covered stent. Conventional stent for jailing the branch with perforation is not recommended because blockage by conventional stent is unreliable and unsuccessful blockage by conventional stent makes subsequent management more difficult.

### Heparin reversal

Heparin reversal is necessary in approaches that require thrombosis. Therefore, heparin reversal should be considered when using a microcatheter or a balloon. Heparin reversal is not generally required when using coils or covered stents. In other management approaches, heparin reversal should be performed as needed, depending on the situation.

### Post-intervention assessment

In the presence of hematoma that does not communicate with the pericardial space, a minimal amount of contrast media via microcatheter should be administered to confirm the absence of hematoma enlargement. If a hematoma is visualized, but its size remains unchanged over time; it indicates that the hematoma pressure is controlled under the resistance of the surrounding tissues, and no further intervention is necessary. If the contrast within the hematoma rapidly fades or expands immediately after contrast injection, this suggests the inflow of blood without contrast media and indicates the possibility of hematoma enlargement over time. Additional interventions are needed in this situation. If communication with the pericardial space has already been established, the cessation of blood outflow into the pericardial space is necessary. There may be multiple routes of blood inflow into the hematoma. In addition to the pre-existing collateral circulation, a hematoma may communicate with nearby vessels as it enlarges. In cases of delayed detection or hematoma enlargement caused by insufficient reduction of hematoma pressure, operators should consider to perform coronary angiography in untouched coronary arteries, to which operators did not advance the guidewires. If blood flow into the hematoma is confirmed, the operator should take appropriate actions.

[Summary]

In the event of guidewire perforation, the risk of cardiac tamponade should be assessed immediately. If there is a significant risk of cardiac tamponade, interventions such as coil embolization and detailed post-intervention assessments can prevent the development of cardiac tamponade. Early detection and prompt management are important to reduce the risk of cardiac tamponade.

## Coronary artery rupture

[Mechanism]

Unlike coronary artery perforation caused by guidewires, coronary artery rupture occurs due to significant damage to the adventitia and perivascular tissues of coronary artery. The main causes of coronary rupture are “overexpansion of coronary artery adventitia and perivascular tissues by balloon and stent expansion” or “damage to the coronary artery adventitia and perivascular tissues by atherectomy devices such as rotational atherectomy, orbital atherectomy, or directional coronary atherectomy.” In CTO PCI, when operators advance guidewires outside the vessel without recognition, operators would advance larger devices such as microcatheters, balloons, or IVUS via the guidewires, which would cause significant damages to the coronary artery adventitia and perivascular tissues and result in coronary artery rupture. Coronary artery rupture is classified as one that involve extensive damage to the perivascular tissues with communication with the pericardial space (Ellis classification Type-3 or Blowout type) and as one that involve partial damage to the perivascular tissues without communication with the pericardial space (Ellis classification Type-2) [11]. In the latter case, even if the hematoma is initially localized within the myocardium, hematoma may continue to expand and eventually rupture into the pericardial space, which may result in cardiac tamponade if the intra-hematoma pressure remains higher than the surrounding tissue resistance. Furthermore, even if cardiac tamponade does not occur, pseudoaneurysm may develop because of the absence of coronary artery adventitia, which warrant careful follow-up.

[Incidence and high-risk situations]

**Fig. 5:**
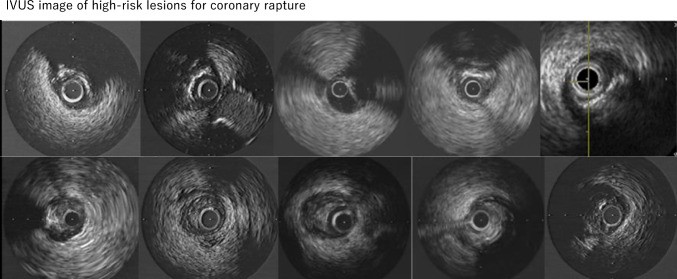
IVUS image of high-risk lesions for coronary rupture. IVUS image of a coronary rupture lesion (upper panels, calcified lesions; lower panels, fibrotic lesions). All lesions were highly eccentric and no plaques were observed on the opposite side

Although comprehensive data are lacking, the reported incidence of coronary artery rupture ranges from 0.2 to 0.5% [9, 10, 12], which is considered to be quite low. Coronary artery rupture due to balloon or stent expansion is more likely to occur when the adventitia is overexpanded in eccentric lesions (Fig. 5). With atherectomy devices, coronary artery rupture is more likely to occur at bend, calcified segment, or area with vessel diameter discrepancies.

[Prediction and confirmation by IVUS]

Among the mechanisms of coronary artery rupture, “overexpansion of coronary artery adventitia by balloon or stent expansion” can be predicted by IVUS, and “guidewire outside the vessel” can be confirmed by IVUS. In terms of the elasticity of coronary artery adventitia, experiments using porcine coronary artery adventitia suggests considerable elasticity (1.8-fold length at 150 mmHg) [13]. Because balloons or stents with a size of 1.8 times the target vessel diameter are not typically used, the risk of coronary artery rupture is low when the coronary artery adventitia is expanded uniformly. However, in eccentric lesions, in which a part of coronary artery adventitia is expanded non-uniformly, rupture due to adventitial overexpansion can occur following balloon or stent dilatation. Therefore, the risk of coronary artery rupture should be assessed and predicted by evaluating the risk of adventitial overexpansion using IVUS before balloon or stent dilatation.

**Fig. 6:**
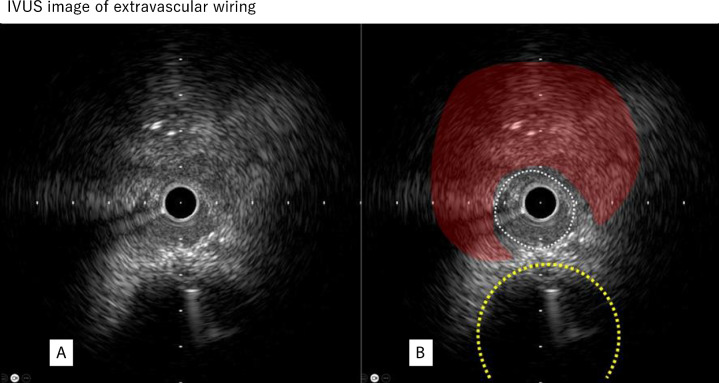
IVUS image of extravascular wiring. **A** IVUS image of extravascular wiring. **B** The white dotted area on the right is a space created outside the vessel, and the area indicated in red is hypoechoic compared with the healthy perivascular tissue, strongly suggesting an extravascular hematoma. The dotted yellow line indicates the original coronary artery

Figure 6A shows the IVUS image obtained when the guidewire and microcatheter were advanced outside the vessel. This finding is characterized by the absence of three-layered structure and the presence of extravascular hematoma. In this situation, the size of damage to the coronary artery adventitia is equal to or greater than the outer diameter of the IVUS catheter. This is a dangerous situation, because the removal of IVUS catheter can cause extravascular blood leakage and potentially cause cardiac tamponade. Since it is rare opportunity to view IVUS images of the guidewire placed outside the coronary artery, it is important to thoroughly understand and recognize the image shown in Fig. 6A, which would help operators respond the situation appropriately when such an image is obtained during PCI. It is also important to recognize the images of extravascular hematoma in IVUS (Fig. 6B). The presence of extravascular hematoma indicates that the coronary artery adventitia is severely damaged or disrupted. When coronary artery damage is suspected, operators should pay attention to the presence of extravascular hematoma during IVUS observation.

[Management: blowout-type coronary artery rupture]

**Fig. 7:**
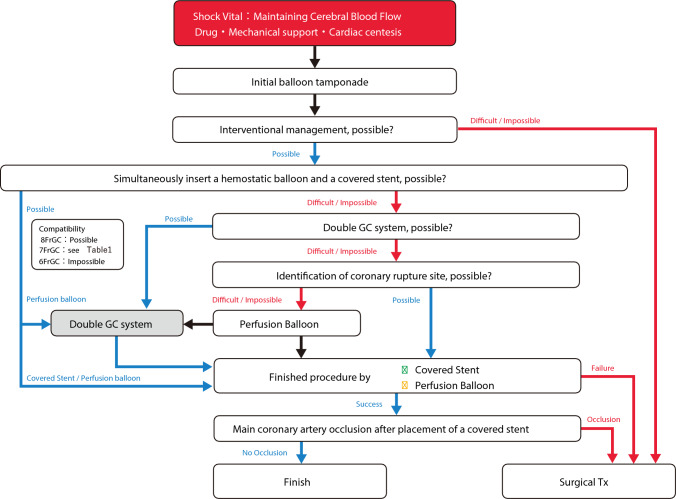
Flowchart for blowout-type coronary artery rupture

In this document, the management of blowout coronary artery rupture is discussed as follows. A flowchart of this process is shown in Fig. 7.

### Initial management

In addition to assembling staff members, calling cardiovascular surgeons, and controlling bleeding from rupture sites, it is critically important to secure “cerebral blood flow.” It is well known that ischemic tolerance is lower in brain than in heart. In cases with poor cardiac output and insufficient cerebral blood flow, “chest compression “and “mechanical support” should be prioritized to the maximum extent.

### Identification of coronary artery rupture site

After securing cerebral blood flow, the rupture site should be identified. In coronary artery rupture, since contrast media flow into the perivascular tissues and the communicated pericardial space, it is often difficult to identify the rupture site. If the rupture site is difficult to identify, operators should try to identify the rupture site by moving the balloon or perfusion balloon used for hemostasis step by step with administering contrast media. If possible, double GC system allows operators to control hemostasis and to identify the rupture site simultaneously.

### Hemostasis procedure

After the coronary artery rupture site is identified and a conventional balloon is used for initial hemostasis, it is reasonable to advance a covered stent along with the already used conventional balloon for hemostasis via the same guide catheter if possible. In this system, switching the conventional balloon to the covered stent within the coronary artery can achieve hemostasis with avoiding the risk of cardiac tamponade. Even when the simultaneous insertion of a conventional balloon and a covered stent is difficult, the simultaneous insertion of a conventional balloon and a perfusion balloon should be considered. If the simultaneous insertion of a conventional balloon and a perfusion balloon is possible, double GC system can be established by switching the conventional balloon to the perfusion balloon with avoiding the risk of cardiac tamponade. Once double-guide catheter system is established, covered stents can be placed while avoiding cardiac tamponade [14]. Table 1 shows the results of a bench test regarding the simultaneous insertion compatibility of hemostatic system with perfusion balloons or covered stents. These results are based on the ability to simultaneously insert devices within the guiding catheter in a dry setting. Because device sizes, guiding catheter conditions, and coronary lesion characteristics were not considered in this bench test, information in Table 1 should be used only as a guide. In the simultaneous insertion of conventional balloon and perfusion balloon or covered stent into a GC, the guidewire of the conventional balloon may limit the advancement of the perfusion balloon or covered stent. If the guidewire used to advance the perfusion balloon or covered stent is securely inserted into the distal true lumen of coronary artery, the removal of the guidewire of the conventional balloon may be effective in this situation.

**Table 1:**
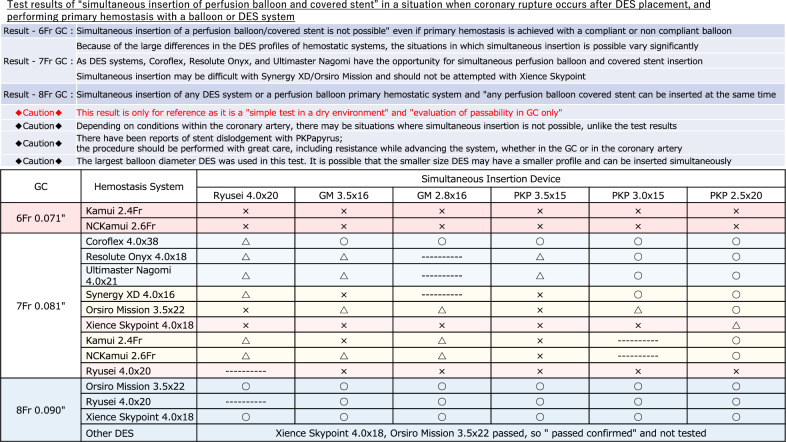
Test results of “simultaneous insertion of a perfusion balloon and covered stent”

**Fig. 8:**
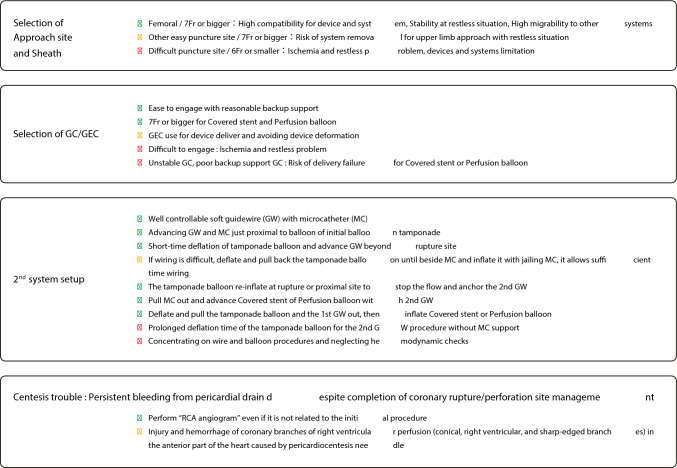
Procedure steps of double GC system/pericardiocentesis trouble

In CTO PCI, ischemia due to prolonged inflation of the conventional balloon is not an issue. Thus, it is possible to establish double GC system while maintaining hemostasis. After establishing double GC system (or if simultaneous insertion of conventional balloon and perfusion balloon or covered stent is possible with the initial single-guide catheter system), the conventional balloon should be exchanged for the perfusion balloon or covered stent within the coronary artery. It is necessary to shorten the duration of hemostatic release as much as possible to avoid cardiac tamponade. When inflating a conventional balloon, the guidewire for the perfusion balloon or covered stent should be advanced near the rupture site. During a brief release of hemostasis, the guidewire should be passed through the rupture site and advanced into the distal true lumen. Microcatheter support may be effective for the guidewire passage while controlling bleeding. If the guidewire passage through the rupture site is difficult, it is useful to advance the microcatheter just proximal to the rupture site and to inflate the conventional balloon outside the microcatheter, which allows operators to manipulate the guidewire while maintaining hemostasis (Fig. 8).

If simultaneous insertion of the hemostatic device and perfusion balloon or covered stent is impossible and double GC system cannot be established, the conventional balloon is removed and exchanged for a covered stent (if the rupture site is clearly identified) or exchange for a perfusion balloon (if the rupture site is unclear). In either case, since it takes substantial time for device exchange (even if performed quickly), significant bleeding can occur, which poses a risk of cardiac tamponade. In this situation, sufficient preparations such as vasopressors and pericardiocentesis should be performed before the conventional balloon is removed.

### Covered stents

Coronary artery rupture involves the disruption of arterial adventitia. Even if temporary hemostasis is achieved with balloon or perfusion balloon, such hemostasis relies on thrombosis within the perivascular tissues. Therefore, there is a possibility of rebleeding due to thrombolysis. Delayed tamponade caused by thrombolysis is also reported in literatures [15, 16]. Therefore, even if hemostasis is achieved with balloon or perfusion balloon, covered stent placement should be considered in case of coronary artery rupture. When placing a covered stent, more careful procedures are required compared to regular stent placement in terms of crossability, stent dislodgement risk, and cover layer damage risk. Crossability is of particular concern in covered stents with double layer of stent struts (Graftmaster: Abbott Cardiovascular, Plymouth, USA). Stent dislodgement and cover layer damage are concern in covered stents with single layer of struts (PK Papyrus: Biotronik, Berlin, Germany). GEC is useful to resolve the weak points of covered stents such as poor crossability, dislodgement risk, and damage risk [17]. However, since covered stents have larger profile than regular stents, it is necessary for operators to know which GECs are compatible with each covered stent. In our bench test, simultaneous insertion of conventional balloon and GEC is only possible with the combination of 8 Fr GC (0.090″) and GuidePlus 5 Fr (Nipro, Settsu, Japan). However, since the inner lumen of GuidePlus 5 is small, it is difficult to bring existing covered stents via GuidePlus 5Fr. Therefore, it is impractical to try simultaneous insertion of conventional balloon and GEC for covered stent delivery.

### Heparin reversal

In principle, when placing a covered stent, “heparin reversal should not be performed.” This is because “thrombosis and coronary occlusion” due to heparin reversal can result in a more serious situation. If heparin reversal is performed for any reason, ACT should be controlled to approximately 150–200, and periodic saline flushing is necessary to prevent thrombus formation within the coronary artery and guide catheter.

### Surgical management

If the coronary artery rupture site is correctly identified and covered stent placement is performed without causing significant coronary occlusion or ischemia, surgical management is unnecessary. Conversely, surgical treatment is required if the coronary artery rupture site cannot be identified, if bleeding cannot be controlled with conventional balloon, perfusion balloon, or covered stent, or if significant ischemia occurs following covered stent placement. When surgical treatment becomes necessary, “information sharing with the surgical team” is extremely important, although time is limited in emergent situations. “Multidisciplinary treatment utilizing all options for surgical and interventional treatment “is essential to overcome this critical situation.

### Non-blowout-type coronary artery rupture

Since patient’s hemodynamics is often maintained in non-blowout coronary artery rupture, more time is available for the bailout procedure than blowout coronary artery rupture. However, because non-blowout-type coronary artery ruptures can progress to blowout-type over time, prompt covered stent placement or perfusion balloon inflation (conventional balloon inflation is also possible in the absence of ischemia) is necessary. Furthermore, it is important to recognize that coronary artery rupture involves the disruption of arterial adventitia even in non-blowout-type coronary rupture. Even if hemostasis is achieved with perfusion balloon or other means, there is a risk of delayed rebleeding due to thrombolysis. Therefore, covered stent placement should be considered depending on the situation.

### Coronary artery rupture during guidewire manipulation in CTO PCI

In coronary artery rupture during guidewire manipulation for CTO PCI, plaque sealing should be attempted first. The coronary artery rupture site can be sealed with plaque by advancing a guidewire, either in antegrade or retrograde manner, into a plaque different from the coronary artery rupture site or into the subintima on the opposite side of the coronary artery rupture site, followed by balloon dilation or stent placement. In this case, creating a dissection in the sealing plaque may establish communication between the lumen and the rupture site, which makes hemostasis difficult. Therefore, smaller balloons and stents should be selected and gradually expanded from low pressure. When the guidewire passes through a subintimal route, including the coronary artery rupture site, and balloon dilation or stent placement is performed along with the subintimal route, there may be cases in which plaque sealing is unsuccessful and bleeding persists. In this situation, it is an option to perform long inflation with perfusion balloon for thrombotic hemostasis or to deploy covered stent. If both plaque sealing and guidewire passage are unsuccessful, the placement of coils in the subintimal space may control bleeding. This situation differs from coil procedures in coronary perforation, because this coil placement is performed in a relatively large lumen, often requiring “longer coils” and “more coils.”

### Persistent bleeding after pericardiocentesis

Despite appropriate management of the coronary artery rupture site and confirmed hemostasis, bleeding from pericardiocentesis drainage may persist in rare cases. In such cases, “RCA angiography” should be performed, because the pericardiocentesis needle might damage the vessels in the RCA territory on the heart surface (Fig. 6b).

### Summary

The most important aspect of bailout for coronary artery rupture is to preserve patient’s life and higher brain function. To achieve this, the first crucial step is to maintain cerebral blood flow. Once cerebral blood flow is maintained, interventionalists and cardiovascular surgeons should provide the best possible treatment.

## Guidewire entrapment/Guidewire fracture

[Mechanism]

Guidewire is an essential device in PCI. Different types of guidewires are available such as workhorses, soft-tip guidewires for frontline use, and stiff-tip guidewires, which are often used in CTO PCI. The development of novel techniques and improvement of interventional equipment in recent years have resulted in a significant increase in PCI success rates and enabled the treatment of more complex lesions. However, there is a risk of complications, particularly when treating CTO, bifurcation lesions, and heavily calcified tortuous vessels. Guidewire entrapment or fracture is more likely to occur during PCI for CTO, heavily calcified and tortuous vessels, in-stent restenosis, or bifurcation lesions accompanying the jailed wire technique [18]. Guidewires left within the vessels, especially if the outer coil is unraveled, are highly thrombogenic, leading to coronary or systemic thrombosis.

[Incidence]

The incidence of guidewire entrapment or fracture is approximately 0.1–0.2% for all PCI [19] and 0.5% for CTO PCI [20]. Guidewire entrapment or fracture is an uncommon but potentially life-threatening complication.

[Causes of guidewire entrapment or fracture]

The causes of guidewire entrapment or fracture include wiring in the CTO, calcified lesions, tortuous lesions, bifurcation lesions, ISR lesions, stent jails, and use of polymer jacket guidewires. The entrapment of the guidewire tip is caused by the tip entering small vessels or vessel spasms. Guidewire ruptures easily when rotational maneuver is applied with an entrapped tip. Additionally, excessive pulling force on the entrapped guidewire can lead to guidewire fracture [21, 22]. This is likely to occur when a knuckle-shaped polymer-jacketed wire is advanced inside the CTO.

There is also a risk of guidewire fracture during procedures such as rotational atherectomy, orbital atherectomy, and directional coronary atherectomy, in which the device rotates at high speed on the guidewire. If the burr or crown advances to the guidewire stopper, or if the device is activated with the guidewire tip trapped within the coronary artery, the guidewire is easily fractured.

Guidewire is composed of inner coil (core) and outer coil. The outer coil is densely wrapped around inner coil in counterclockwise direction. The flexibility of the guidewire tip is ensured by thinning the core, where the distal 3 cm of the tip is radiopaque. There are various types of “fracture,” such as detachment of flexible tip, fracture of core, and unraveling or stretching of outer coil. Although detachment of the flexible tip is unlikely to cause coronary injury, fracture of the guidewire shaft can cause arrhythmia, coronary dissection, or perforation [4, 23]. Additionally, if the outer coil is unraveled, the outer coil becomes thin and flexible, forming a nidus with high thrombogenicity. If the unraveled outer coil remains in the coronary artery, it may cause coronary thrombotic occlusion, and if the unraveled outer coil remains in the aorta, it may cause systemic embolization [24].

[Prevention]

Guidewire should not be rotated excessively, and the clockwise and counterclockwise rotations should be maintained until approximately 180 degree. Furthermore, when the tip is trapped, rotational maneuver should not be applied. It is particularly important to check the mobility of the guidewire tip during rotational atherectomy, orbital atherectomy, and directional coronary atherectomy. In clinical practice, guidewire is commonly used to protect the side branches during stent deployment, and it is sometimes difficult to remove the jailed guidewire. It is important to remove the jailed guidewire before high-pressure post-dilatation, to be particularly careful in long stent for calcified lesions, and not to jail the radiopaque part with stent. The use of long stent in heavily calcified lesions is a risk factor for guidewire entrapment or fracture because the guidewire is tightly sandwiched between the stent and calcified plaque. Recently, special techniques such as jailed corsair technique [25] and jailed balloon technique [26] have been developed to protect the side branches when treating bifurcation lesions. Although these procedures are intended to protect side branches, these procedures also protect the guidewire, which prevents guidewire entrapment or fracture in bifurcation lesions.

[Management]

**Fig. 9:**
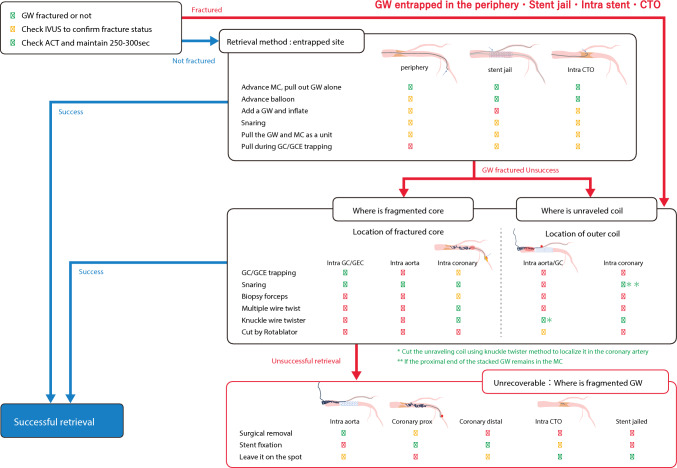
Flowchart for guidewire entrapment or fracture. CTO, chronic total occlusion; GEC, guide-extension catheter

There are three potential approaches for managing guidewire entrapment or fracture: (1) percutaneous removal, (2) surgical removal, and (3) conservative management, which leaves the fragment in coronary artery. Removal of the guidewire fragments is recommended in most cases and should ideally be achieved using percutaneous techniques, although surgical removal may be required in some cases. Management of these complications is predominantly based on the operator’s experience and knowledge. Therefore, we proposed a structured algorithm for managing guidewire entrapment and fractures. A flowchart of the procedure for treating guidewire entrapment is shown in Fig. 9.

[Percutaneous removal]

### Microcatheter method

The microcatheter should be advanced over the entrapped wire as closely as possible to allow release of the jailed or entrapped part [4, 27]. Rotative microcatheters such as Corsair (Asahi Intecc, Seto, Japan) or Tornus (Asahi Intecc, Seto, Japan) may be effective in passing through the jailing part and eventually freeing the entrapped guidewire. If the GW is covered by the microcatheter, the use of a snare is also an option, as described below.

### Plaque modification method

The balloon should be advanced as far as possible over the entrapped guidewire and inflated, potentially freeing the wire from the surrounding tissue [18]. A small-profile balloon is recommended for entrapped jailed guidewires. Alternatively, a second guidewire may be advanced around the entrapped guidewire followed by balloon inflation to disengage the guidewire from the coronary artery wall.

### Guide catheter/GEC trapping method

If the wire remains intact, or the fractured core remains within the guide catheter or GEC, a conventional balloon is advanced and inflated at the terminal part of the guide catheter or GEC. The entire system is then retracted to remove the retained or fractured guidewires as one unit [19].

### Multiple guidewires twisting method

This method is also valid for guidewire fractures. Two or more guidewires should be advanced alongside the entrapped guidewire, with a torquer device applied to all guidewires. A twisting action results in the guidewires wrapping around the retained guidewire, eventually trapping the fractured fragment between the wrapped portions [28]. The twisted group is then retracted, pulling the entrapped guidewire out of the coronary artery toward the guide catheter as a whole.

### Knuckle-twister method

**Fig. 10:**
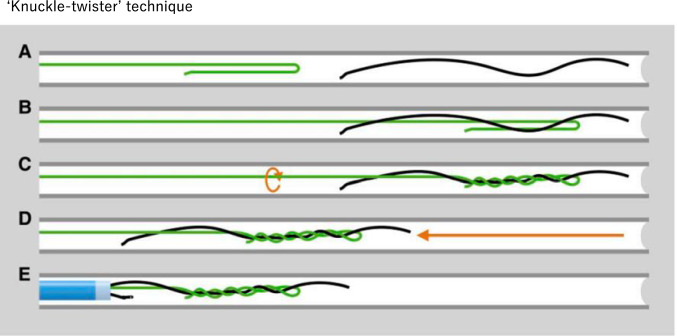
The “Knuckle-twister” technique. This method retrieves a fractured or entrapped guidewire by forming the tip of the polymer-jacketed guidewire into a knuckle of 3–6 cm (**A**), advancing it slightly distal to the lost wire (**B**), and pulling it back while continuously drilling until the wire becomes completely entangled with the lost wire (**D, E**). This figure has been reprinted with permission (Leibundgut et al. [29])

Similar to the guidewire twist method, the knuckle-twister method is useful when guidewire fractures occur [28, 29]. A polymer-jacketed tapered guidewire such as Fielder XT-A (Asahi Intecc, Seto, Japan) is used as a retrieval guidewire. A knuckle of ∼3–6 cm in length (Fig. 10A) should be formed and advanced slightly distal to the lost guidewire (Fig. 10B). Thereafter, the drilling of the knuckle guidewire is commenced (Fig. 10C). A pullback is performed while drilling continuously until the guidewire becomes completely entangled with the lost guidewire (Fig. 10D, E). The entanglement formed by knuckle-twister technique is notably tighter and stronger than entanglement formed by multiple guidewire twisting technique. This method can be used to cutting as well as removal for unraveled outer coils.

### Snaring

Grasping the entrapped guidewire with a snare allows operators to pull out the entrapped guidewire with strong force. However, there is a possibility of tearing or damaging the guidewire, making subsequent procedures difficult. A micro-loop snare with a loop diameter of 2 or 4 mm is advanced as much as possible over the entrapped guidewire and tightened to facilitate guidewire retrieval. The snare is loaded from the tail of guidewire and advanced coaxially to the distal tip, where the snare is closed and pulled together with the entrapped guidewire [30]. EN Snare (MERITMEDICAL, South Jordan, USA) has three loops that increase the possibility of retrieval. In cases where remnant fragments remain in the distal coronary artery, Soutenir (Asahi Intecc, Seto, Japan) should be considered for its excellent derivability, even to the far distal parts of coronary artery.

If the proximal part of the entrapped guidewire remains within a microcatheter (GW is covered by the microcatheter), it is recommended to use 4 mm snare via the microcatheter after cutting the hub of the microcatheter. Even the 6Fr guide catheter can accommodate 4 mm snare and the small-diameter microcatheter simultaneously. This method allows operators to bring the snare until the entrapped guidewire easily. Note the combination of snare and small-diameter microcatheter is recommended rather than the snare system. In a bench test for this document, it was possible to cut or retrieve the entrapped guidewire.(In this case, 4 mm Goose Neck Snare (Medtronic, Dublin, Ireland) + Finecross GT (Terumo, Tokyo, Japan) was used). Because it is necessary to remove the microcatheter in multiple guidewires twisting method or knuckle-twister method, this method may be considered as the first choice before trying to multiple guidewires twisting method or knuckle-twister method.

In Fig. 9, the first step is to verify whether the guidewire is fractured. Next, the continuity of the guidewire core should be confirmed using fluoroscopy. If it is difficult to determine the continuity with fluoroscopy, IVUS is useful. To prevent thrombotic complications, additional heparin should be considered to maintain ACT of 250–300 s or more. If the entrapped guidewire is intact (blue line), the first-line treatment is to insert a microcatheter and remove the entrapped guidewire. Next, a small-profile balloon is inserted along the entrapped guidewire and inflated to free the entrapped guidewire from the surrounding tissue, as described by the plaque modification method. In addition, guide catheter/GEC trapping and snaring methods should be considered. Finally, the microcatheter and the guidewire are removed together. Based on the guidewire entrapment or fracture status, the flow described in green should be considered first, followed by yellow. Red indicates relatively risky methods. If the guidewire is fractured initially or during the retrieval process, proceed to “How to retrieve a fractured guidewire’’ on the chart. Usually, “guidewire fracture’’ refers to a fracture of the core, and the outer coil is often left behind and unraveled. Furthermore, the unraveling of the outer coil is difficult to confirm using fluoroscopy and can only be confirmed by IVUS. The chart indicates whether the proximal end of the fractured core is within guide catheter or GEC, aorta, or coronary artery, regardless of the presence or absence of outer coil unraveling, as indicated by green, yellow, and red, respectively. If the guidewire core remains in the guide catheter/GEC, the guide catheter/GEC trapping method is used to target the fragmented core's proximal end. If the guidewire core remains in the coronary artery, the guide catheter/GEC should be advanced into the coronary artery, and the guide catheter/GEC trapping method should be attempted. However, additional attention should be paid to coronary injuries. Snaring, biopsy forceps, or Soutenir may be used to grasp the proximal end of the fragmented core. During retrieval, the grasped proximal end of the guidewire may cause coronary artery injury. The use of GEC may reduce this risk of coronary artery injury. If strong resistance is observed within the coronary artery during retrieval, it should not be forcefully removed. In this situation, stent fixation within coronary artery should be considered [21, 23, 27]. If the fragmented core floats in the aorta, snaring is attempted by using a large Goose Neck Snare or a handmade snare. To prevent aortic injury, the part gripped by the snare should be as close as possible to the proximal end of the remaining core. The complete fracture of a flexible tip or ball tip can remain within the coronary artery. If the tip is not mobile (fixed) within CTO or small vessels, it may be unnecessary to retrieve the tip. If the fragmented core is mobile within the coronary artery, the guidewire twist or knuckle-twister method should be used to retrieve the tip. If the outer coil is unraveled and left behind, several options may be considered depending on whether the unraveled outer coil “extends to the aorta” or “is localized within the coronary artery.” However, it is difficult to manage the remnants of an unraveled outer coil. This is not only because the remaining outer coil exhibits high thrombogenicity but also because snare gripping or guide catheter trapping methods is ineffective, which can cause further distraction of the outer coil. (As mentioned above, if the proximal part of the entrapped guidewire remains within a microcatheter, it is recommended to use 4 mm snare via the microcatheter after cutting the hub of the microcatheter.) The bailout procedure aims to cut the unraveled outer coil and localize it in the coronary artery. The knuckle-twister method is an option. Once the outer coil is cut and localized in the coronary artery, the shortened outer coil can be fixed with a stent. Depending on the operator’s technique, rotational atherectomy may be useful to cut the remnant outer coil at the ostium of the coronary artery [31]. If serial attempts fail and the outer coil remains in the aorta, there is a possibility of systemic embolization depending on its length. Therefore, surgical removal must be considered.

[Summary]

Guidewire entrapment or fracture is a rare but potentially life-threatening complication. Although percutaneous retrieval is an ideal bailout, it is necessary to consider the possibility of subsequent complications caused by prolonged percutaneous retrieval, such as coronary artery occlusion, dissection, or perforation. Rather than pursuing complete retrieval, alternative options such as stent fixation on the coronary artery wall, surgical removal, and conservative treatment should be considered to save patient’s life. On the other hand, high mortality is expected in emergent surgical removal combined with coronary artery bypass graft surgery. Appropriate treatment should be selected for each patient and each type of guidewire fracture.

## Imaging device entrapment

[Mechanism]

**Fig. 11:**
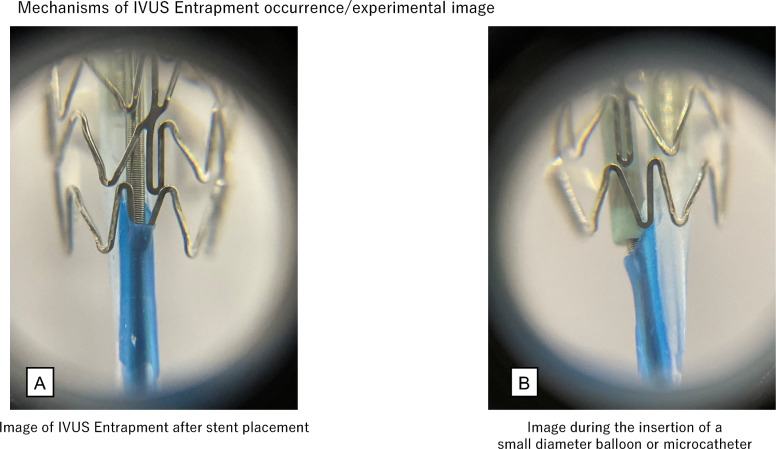
Experimental image of the mechanisms of IVUS entrapment occurrence. Experimental image of IVUS entrapment after stent placement (**A**). Experimental image during the insertion of a small-diameter balloon or microcatheter (**B**). The device tip occluded the exit port, leaving no space for entrapment of the stent strut

**Fig. 12:**
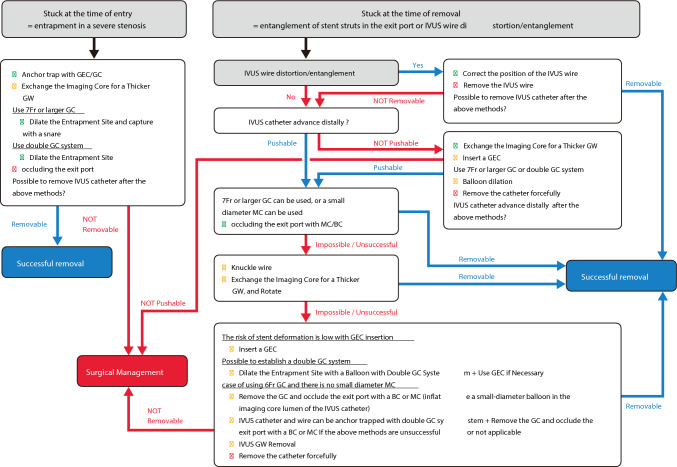
Flowchart for imaging entrapment

IVUS catheter entrapment can occur because of the impaction of stent strut into the exit port of IVUS catheter (Fig. 11A), excessive pushing of the IVUS catheter into the severe stenosis, or entrapment associated with guidewire bending or kinking. Naturally, this complication can occur not only with IVUS catheters but also with OCT and optical frequency domain imaging (OFDI) catheters. Imaging catheter entrapment can occur in both short monorail and wire-rail types that have a second guidewire lumen. Management varies depending on causes of entrapment. A flowchart of this process is shown in Fig. 12. In cases of entrapment in severe stenosis, operators should try to remove the catheter by dilating the lesion or, if possible, to grasp the entrapped device as close to the entrapment site by snare. If the cause of entrapment is related to the guidewire, operators should try to remove the IVUS catheter after advancing the IVUS catheter to correct the guidewire bending and slowly withdraw the IVUS catheter together with the guidewire. This document focus on the impaction of stent strut into the exit port of IVUS catheter, which is the most fundamental issue in IVUS entrapment.

[Predisposing conditions]

Regarding the impaction of stent strut into the exit port, Hiraya et al. reported that the incidence of IVUS entrapment is high in cases with very tortuous lesions, severely calcified lesions, third-generation drug-eluting stents, and stent diameters of ≤ 2.5 mm [32]. The characteristic feature of the exit port entrapment, which is different from other types of IVUS entrapment, is that it is easy to push the IVUS catheter distally after the occurrence of IVUS entrapment, but the catheter becomes entrapped at the same location when the IVUS catheter is pulled back.

[Management]

Forceful pulling the IVUS catheter would make the entrapment more rigid and make it impossible to push the distal portion of the catheter, which is more difficult situation to overcome. For the prevention of the rigid entrapment, the IVUS catheter should be pulled back under fluoroscopy. If entrapment occurs, it should not be pulled back forcefully. If the mechanism of entrapment is the impaction of stent strut into the exit port, entrapment can be avoided by occluding the exit port or by misaligning the position of the exit port and stent struts when pulling the IVUS catheter back.

### IVUS catheter rotation

If the IVUS catheter advances distally, rotating the IVUS catheter and pulling it back again may resolve the entrapment. However, since this method is unreliable, it is important not to stick to IVUS catheter rotation.

### Exit port occlusion

If entrapment cannot be resolved by rotating the IVUS catheter as initial procedure, the exit port occlusion technique should be performed. The operator needs to advance the IVUS catheter distally to occlude the exit port. A microcatheter or balloon catheter is inserted over the guidewire, which is used to advance the IVUS catheter, and then the microcatheter or balloon catheter is advanced until the tip contacts the exit port (Fig. 11B). The IVUS catheter is then removed by pulling it back with the device. In this case, if the microcatheter or balloon catheter used to occlude the exit port is pulled back first, occlusion of the exit port may become insufficient and the IVUS catheter may become entrapped again. Maintaining occlusion of the exit port is important while pulling back the IVUS catheter and the device. This exit port occlusion technique is highly reliable. However, there are limitations for its implementation. If the guide catheter is ≥ 7 Fr, a slimmer microcatheter or balloon catheter can be selected and advanced through the guide catheter along with an entrapped IVUS catheter. However, if the guide catheter is 6 Fr or smaller, the devices that can be used in combination with the IVUS catheter are limited depending on the IVUS catheter used. Information regarding the compatibility between the IVUS catheters and combinable devices should be obtained in advance. The conditions for the simultaneous insertion of the imaging catheters and microcatheters or balloon catheters when using 6 Fr guide catheter are as follows: If the outer diameter of the IVUS catheter shaft is 3.0–3.2 Fr, insertion of 1.9 Fr microcatheter is possible. However, if the outer diameter is 3.6 Fr, even with 1.6 Fr microcatheter, there is a strong resistance or difficulty in insertion; therefore, this is not currently recommended. The results of the bench test conducted by this task force are shown as reference information. In 6 Fr guide catheter, simultaneous insertion of IVUS catheters including “OptiCross (Boston Scientific, Marlborough, USA): tip 2.4 Fr, exit port 3.15 Fr, proximal 3.0 Fr,” “AltaView (Terumo, Tokyo, Japan): tip 2.6 Fr, proximal 3.0 Fr,” “AnteOwl (Terumo, Tokyo, Japan): tip 2.6 Fr, proximal 3.1 Fr,” and 1.9 Fr Carnelian Marvel Micro Catheter non Taper (Tokai Medical Products, Kasugai, Japan) was possible. In 6 Fr guide catheter, simultaneous insertion of “Dualpro (Infraredx, Bedford, USA): tip 2.4 Fr, exit port 3.2 Fr, proximal 3.6 Fr” and Carnelian Marvel Micro Catheter (Model: S MXNS155X 1.6/1.8 Fr, 155 cm single marker: Tokai Medical Products, Kasugai, Japan) was difficult, which was judged not to be recommended. When using Dualpro, it may be worth considering using 7 Fr guide catheter. Simultaneous insertion of any IVUS catheters and balloon catheter is difficult in 6 Fr guide catheters.

### Advance entrapped IVUS catheter distally

If the exit port entrapment becomes rigid and the IVUS catheter does not advance distally, the imaging core should be removed and replaced with a thicker hydrophilic guidewire. The IVUS, OCT, and OFDI catheters and exchangeable imaging core guidewires are as follows: AltaView with 0.021″ guidewire; FastView (Terumo, Tokyo, Japan) with dry 0.018″ guidewire (this must be dry without water priming); Dragonfly (Abbott Cardiovascular, Plymouth, USA) with 0.018″ guidewire; OptiCross with 0.025″ guidewire; Dualpro with 0.025″ guidewire. To exchange a thicker guidewire, the hub at the proximal end of the imaging catheter is loosened or removed, or the distal end of the stretchable part of the catheter is cut. This exchange increases the force required to push the IVUS catheter distally, potentially moving the IVUS catheter distally. If the IVUS catheter moves distally and exit port occlusion is possible, the IVUS catheter should be removed by occluding the exit port. If the exit port occlusion technique cannot be performed in the limitation of guide catheter size or if it is unsuccessful, the following procedures should be considered:

### Advance soft 0.014″ guidewire with knuckle shape beside the IVUS catheter

If the IVUS removal route deviates from the stent strut after using knuckle guidewire, the IVUS catheter can be removed. Multiple knuckle guidewire insertions may increase the success rate. However, these results are difficult to control.

### Establish double-guide catheter system, dilate the entrapment site with balloon, and use GEC if necessary

If treatment is performed using single-guide catheter system, another puncture is required and the second guide catheter should be advanced into the same coronary artery. Once double-guide catheter system can be established, operators can dilate the entrapment site via the second guide catheter, which can change the positional relationship between the exit port and entrapped struts. In this situation, high success rate can be expected. Furthermore, balloon dilation from the second guide catheter can be combined by cutting the IVUS catheter and advancing the GEC, as described below.

### Cut the IVUS catheter, exchange the imaging core for a thicker guidewire, and rotate the IVUS catheter if necessary

The insertion of a thicker guidewire may shift the IVUS withdrawal route, potentially allowing the removal of the IVUS catheter possible. The insertion of a thicker guidewire may also improve rotational performance, and the rotation of the IVUS catheter can be used in combination.

### Cut the IVUS catheter and advance the GEC/child catheter

If the tip of the GEC/child catheter can be advanced beyond the entrapment site, the struts will no longer be entrapped in the exit port and the IVUS catheter can be reliably removed. Even if the tip of the GEC/child catheter does not reach the entrapment site, advancing it close to the entrapment site may change the positional relationship between the exit port and struts, potentially resolving the entrapment. However, incomplete stent expansion may cause IVUS catheter entrapment, and there is a risk of stent deformation with the insertion of GEC/child catheter. Stent deformation combined with IVUS entrapment can result in serious complications. Therefore, careful consideration is required to determine whether this method should be performed.

### IVUS catheter cutting, guide catheter removal, exit port occlusion, and anchor trapping with double-guide catheter if necessary

If 6 Fr or smaller guide catheter is used and the abovementioned exit port occlusion cannot be performed, exit port occlusion can be performed by advancing a balloon catheter or microcatheter over the IVUS guidewire after cutting the IVUS catheter and removing the guide catheter. To prevent unintended movement of the IVUS catheter and guidewire during guide catheter removal, in addition to using an extension wire, a 0.014″ guidewire and a small-diameter balloon can be advanced into the imaging core lumen of the IVUS catheter and the balloon can be inflated within the imaging core lumen. This method supports constant holding of the IVUS catheter and guidewire even during guide catheter removal and prevents unintended movement of the IVUS catheter and guidewire. If balloon catheter or microcatheter insertion is difficult after guide catheter removal, double-guide catheter system can be established and the IVUS catheter and guidewire can be anchored distal to the entrapment site. By anchoring the IVUS catheter and guidewire, pulling tension can be applied to the IVUS guidewire while inserting the balloon catheter or microcatheter, which increases the possibility of successful balloon catheter or microcatheter insertion. However, because anchor trapping is performed within the coronary artery (not within the guide catheter), the IVUS catheter and guidewire may be withdrawn unintentionally.

### IVUS guidewire removal

Finally, when other methods are unsuccessful or cannot be applied, the guidewire of IVUS catheter is removed. Removing the guidewire of IVUS catheter may change the positional relationship between the exit port and the struts, allowing the IVUS catheter to be removed. However, caution is required in this option, because removing the guidewire of IVUS catheter makes the exit port occlusion technique unavailable.

### Procedure after successful IVUS catheter removal

After removal of the IVUS catheter, stent expansion and coronary artery blood flow should be confirmed. At this time, since confirmation of stent deformation using intravascular imaging is not usually recommended, it is important to perform post balloon dilatation to adjust the stent struts.

### Surgical management

If all the above procedures are unsuccessful, surgical retrieval should be considered before hemodynamic deterioration. In general, surgical retrieval is safe and straightforward.

## Bailout from the entrapment of rotational atherectomy burr

[Mechanism and incidence]

Rotational atherectomy remains the cornerstone treatment for severely calcified coronary lesions in contemporary PCI [33, 34]. Burr entrapment is a unique complication associated with rotational atherectomy. The incidence of burr entrapment is approximately 0.4–0.8%, based on single-center studies [35, 36]. Several mechanisms have been proposed for the burr entrapment. One such mechanism is the Kokeshi phenomenon. In this phenomenon, the coefficient of friction during motion is smaller than that at rest [36]. Burr entrapment due to the Kokeshi phenomenon may occur after forceful manipulation with small burrs. Vessel angulation is another possible mechanism for burr entrapment. The burr can be entrapped by non-severe calcification at the site of angulation because the burr shape is ellipsoid and the diamond coating is absent at the tail of the burr. Furthermore, the burrs may be entrapped by previously deployed stent struts. Careful manipulation is necessary when the burr advances within or beyond previous stents.

To prevent burr entrapment, operators should recognize changes in the rotational speed, sound of ablation, and resistance during rotational atherectomy. If operators encounter burr entrapment, they must remain calm. Operators should assess the situation, including the presence of antegrade flow, changes in electrocardiogram, and the extent of the patient’s chest pain. In the absence of antegrade flow beyond the burr, percutaneous bailout is expected to be difficult. Operators should contact cardiovascular surgeons and discuss options including surgical bailouts. Furthermore, if the vital signs are unstable, operators should not hesitate to use mechanical support devices. However, if the antegrade flow is maintained without ST-segment elevation, operators can try percutaneous bailout techniques. Before attempting bailout techniques, operators should consider securing additional femoral access (arteries and veins) for the rescue sheaths or double-guide catheter system. Because massive perforation may occur immediately after retrieval of the entrapped burr, rescue sheaths may be useful for establishing mechanical support devices smoothly.

[Management]

**Fig. 13:**
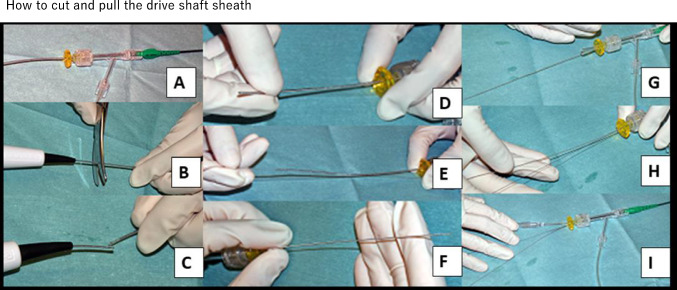
How to cut and pull the drive shaft sheath. **A** An RA burr (1.25 mm) is inserted into a 6 Fr guide catheter via a Y-connector. **B**, **C** The drive shaft, drive shaft sheath, and RA wire are cut together near the advancer. **D**, **E** The drive shaft sheath was pulled back and removed. **F** After the drive shaft sheath is removed, the drive shaft remains in the same position. **G**, **H** A guidewire (0.014″) is passed through the guide catheter via an inserter and Y-connector. **I** A 2.5 × 15 mm conventional balloon easily passed through the guide catheter. This figure was reproduced with permission from Sakakura et al. [37]

Although several percutaneous bailout techniques have been reported in the literature [37–45], the focus among the various bailout techniques is whether to establish double-guide catheter system. If operators select the double-guide catheter system, they attempt to cross the lesion using a workhorse guidewire from the second guide catheter and dilate the proximal segment of the entrapped burr using a conventional balloon [38]. The advantage of double-guide systems is highlighted in the ping-pong technique when massive perforation occurs following burr retrieval [46]. If the Kokeshi phenomenon causes entrapment, advancing the guidewire beyond the burr would be difficult. Guidewires with hydrophilic coating or polymer jacket guidewires may be useful; however, stiff guidewires for the penetration of CTO may be necessary to cross the entrapped burr [40]. Although microcatheters may be necessary to advance the guidewire beyond the entrapped burr, operators should recognize that the 6 Fr or 7 Fr guide catheter cannot accommodate the drive shaft sheath and microcatheter simultaneously. Even if operators select single-guide system, it is recommended to cross the lesion using the workhorse guidewire from the same guide catheter because this guidewire can be a lifeline when massive perforation occurs following burr retrieval. In the single-guide system, the next step depended on the size of the guide catheter (8 Fr or 7 Fr). If operators use an 8 Fr guide system, the lesion is crossed using the workhorse guidewire and then dilation of the proximal segment can be attempted using a conventional balloon. However, if operators use ≤ 7 Fr guide system, they need to cut and pull the drive shaft sheath (Fig. 13) [37] because ≤ 7 Fr guide catheter cannot accommodate the drive shaft sheath, guidewire, and balloon catheter simultaneously. Once the drive shaft sheath is removed, the operator can advance the balloon to the proximal segment. Furthermore, operators can advance GECs via the drive shaft after the retrieval of the drive shaft sheath [39, 42, 43]. Another option is to use a snare within the GEC to hold the drive shaft, which may cause burr disconnection. It is important to recognize that each size of guide catheter can accommodate the drive shaft, microcatheters, balloon catheters, and snare simultaneously, as shown in Table 2. However, Table 2 is based on a bench test, which may differ from real clinical practice. Therefore, the results of this bench tests do not guarantee the successful insertion of each device. Larger guide catheters often offer the advantage of simultaneous accommodation.

**Table 2:**
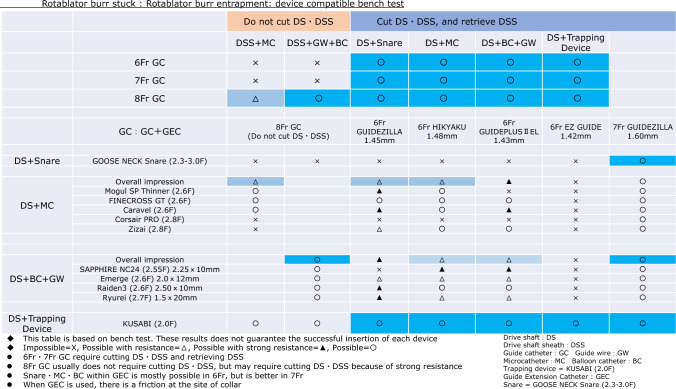
Rotablator burr entrapment: results of simultaneous device insertion tests by removal and non-removal of drive shaft sheaths

**Fig. 14:**
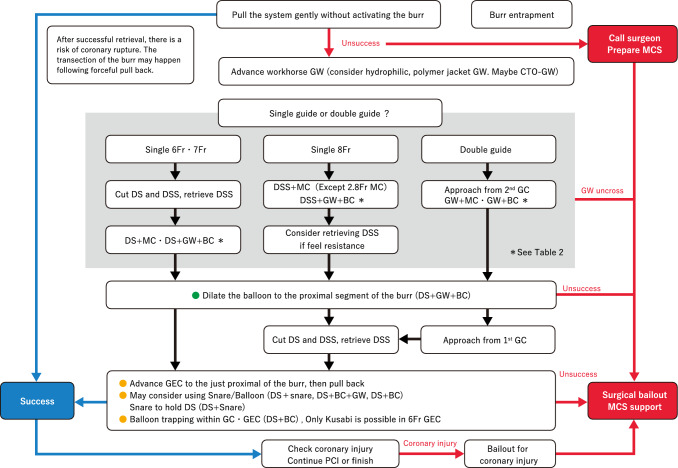
Flowchart for burr entrapment. DS, drive shaft; DSS, drive shaft sheath; GW, guidewire

During the bailout process, operators should recognize the possibility of severe complications including massive perforation and dissection following burr retrieval [47]. It is critical to prepare for complications after burr retrieval. In both the single- and double-guide systems, the workhorse guidewire should be inserted deeply beyond the burr. Operators can insert perfusion balloons or covered stents via the workhorse guidewire when massive perforation occurs after burr retrieval. The prevention and bailout of burr entrapment are summarized in Table 3 [34]. The differences between the single- and double-guide systems are summarized in Table 4. A detailed flowchart of the bailout process is shown in Fig. 14.

**Table 3:** Prevention and bailout for burr entrapment

Prevention or bailout	Concept	Specific methods/comments
Prevention	Risk assessment is of utmost importance for prevention of perforation following RA	Do not push the burr too much, just deliver the burr to the lesion Greater risk in lesions with an angulation Be careful about rotational speed deceleration, sound of ablation, and resistance during the burr manipulation
Prevention	Do not inactivate the burr in the middle of the calcified stenosis	There is no diamond coating at the tail of the burr Moderate stenosis at the proximal of the target can be a cause of burr entrapment
Bailout	It is important to assess the situation such as the presence of antegrade flow, calmly	Do not activate the burr after burr entrapment Bailout techniques are divided to single guide bailout or double guide bailout Contact cardiovascular surgeons immediately in case of unsuccessful percutaneous bailout

This table was reproduced with permission from Sakakura et al. [34]

**Table 4:** Difference between single- and double-guide systems in the bailout for the entrapped burr

	Single guide system	Double guide system
Advantage	Does not require additional arterial puncture Simpler procedures than double guide system	Ping-pong technique can be applicable to manage coronary rupture following the burr retrieval It may be possible to reuse the rotablator system after the bailout
Disadvantage	It may be difficult to manage coronary rupture following the burr retrieval A 6 or 7-Fr system require the retrieval of the drive shaft sheath It is impossible to reuse the system after the retrieval of the drive shaft sheath	Require additional arterial puncture under the full heparization The bailout using guide extension catheter require the retrieval of the drive shaft sheath

## Stent dislodgement

[Mechanism and incidence]

Stent dislodgement from the stent delivery system is a relatively rare complication in currently used stents because of improvements in the crimping force of the mounting balloon, stent delivery performance, and stent delivery technique [3, 48, 49]. However, this is often caused by an attempt to retrieve the stent when the stent fails to pass or becomes stuck owing to severe calcification or bending of the coronary artery. To prevent stent dislodgement, adequate lesion preparation by sufficient dilation of the target lesion and careful stent delivery (not forcing the stent into the target lesion) is essential with clear understanding of moderate stenosis, calcification, and bending toward the target lesion. It is important to use GEC if necessary. It is also important not to apply negative pressure to the mounted balloon until the stent is delivered to the site of implantation.

Despite these precautions, stent dislodgement may occur. In such cases, the outcome depends on whether the stent is severely deformed and whether the guidewire remains inside the stent. Therefore, the first step is to confirm these two points and act as quickly as possible to avoid secondary complications. Various interventional techniques must be used to retrieve a stent trapped in a coronary artery [50]. It is also important to consider whether a stent can be retrieved during each step of the retrieval methods.

### Management

It is recommended to use femoral arteries for stent retrieval. This is because the femoral approach allows operators to use a large-diameter guide catheter for retrieval. Retrieval in the descending aorta would avoid stent migration into the head and neck arteries [51]. If stent dislodgement occurs during the radial artery approach, bailouts should be attempted only if the stent is likely to be retrieved smoothly into the guide catheter, because of the high risk of vascular injury.

**Fig. 15:**
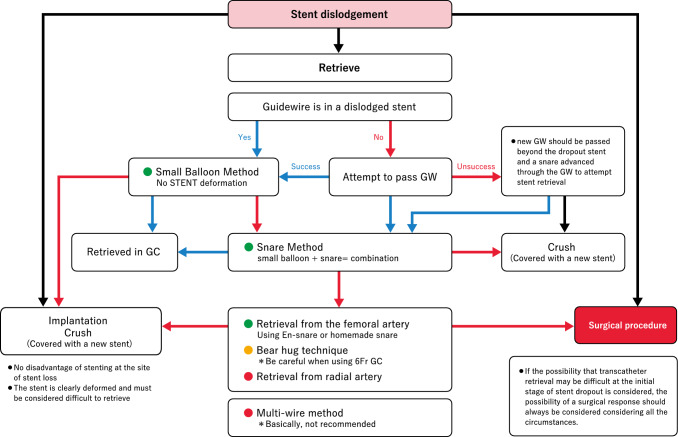
Flowchart for stent dislodgement. Stenting: if there is no obvious stent deformation, there is no significant disadvantage to implanting the stent at the site of stent loss, and if the guidewire is still inside the stent, stenting at the site of stent loss is an option. The surgeon can attempt to insert a small balloon (balloon size 1.5 mm × 10 mm) into the dislodged stent. If successful, the surgeon should increase the balloon size to match the vessel diameter at the implantation site. Stent retrieval. Small balloon method. The operator inserts a small-diameter balloon into the dislodged stent, dilates the balloon at the proximal site, and retrieves it. Snare method. A snare catheter can also be used to retrieve a dislodged stent through the guidewire inside the stent using Medtronic Goose Neck Snares. Small balloon/snare combination. If delivery of a snare is challenging, it can be mounted on a small-diameter balloon to reach the stent site. Multi-wire method. Two additional guidewires are added outside the stent and advanced into another branch on its peripheral side. Thereafter, the three guidewires are placed on a torquer and continuously turned in the same direction. When the stent starts to rotate, it should be moved proximally and retrieved while maintaining rotation. This method is not recommended because of the possibility of stent detachment from the multi-wire and low success rates

In addition, if a thrombus develops around the dislodged stent at the initial stage of stent dislodgement and causes hemodynamic instability, or if the stent is severely deformed, it may be difficult to perform percutaneous retrieval. In this situation, surgical bailout is an alternative option. A flowchart of this process is shown in Fig. 15.

### Stenting

If the stent is severely deformed and retrieval is considered to be difficult, operators should insert a new guidewire to pass beside the deformed stent and expand the balloon and new stent at the site of stent dislodgement to seal the dislodged stent. If there is no obvious stent deformation and the guidewire is still inside the dislodged stent, it is an option to dilate the dislodged stent at the site of stent dislodgement. First, operators can attempt to insert a small balloon like 1.5 mm × 10 mm into the dislodged stent. If successful, operators should increase the balloon size to match the diameter of the vessel at the implantation site. It should be noted that the stent may move distally when the balloon is inserted into the stent, and the final stenting site may be distal to the dislodged site. If the stent can be moved during the insertion of a small-diameter balloon, operators may consider moving the stent into the desirable site.

### Stent retrieval

Operators inserts a small-diameter balloon into the dislodged stent, dilates the balloon at the proximal site, and retrieves it. The balloon should be approximately 1.5 mm × 10–15 mm and dilated at low pressure. Proximal rather than distal balloon dilation is recommended, because a well-designed bench test have shown that proximal dilation is more effective than distal dilation [52]. During proximal dilation, approximately half of the balloon should cover the stent, and the proximal edge of the stent should be in the center of the balloon. When retracting the stent into the guide catheter, it is necessary to confirm the coaxiality of the balloon and guide catheter using fluoroscopy from multiple directions and to fine-tune the position of the guide catheter (height and direction).

A snare catheter can also be used to retrieve a dislodged stent through the guidewire inside the stent using the Goose Neck Snare. The proximal portion of the stent is grasped and slowly pulled back into the proximal portion of the coronary artery. When pulling the stent into the guide catheter, it is crucial to confirm coaxiality with the stent grasped with snare using fluoroscopy in multiple directions and to fine-tune the position of the guide catheter (height and direction).

**Fig. 16:**
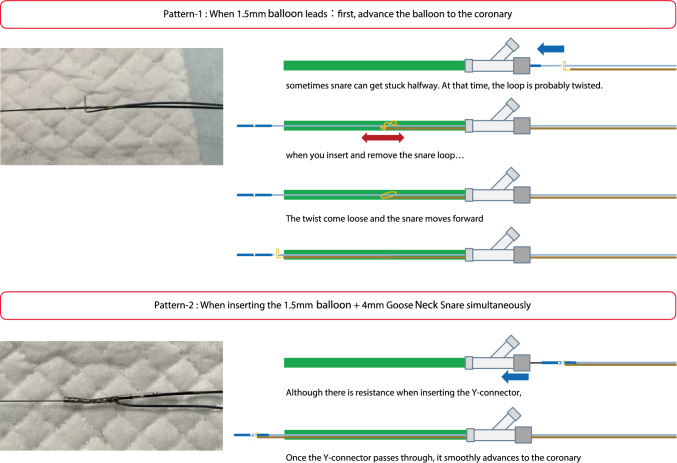
Small balloon with snare. Usually, a Goose Neck Snare is combined with a guidewire and a small-diameter balloon to facilitate reaching the dislodged stent

**Fig. 17:**
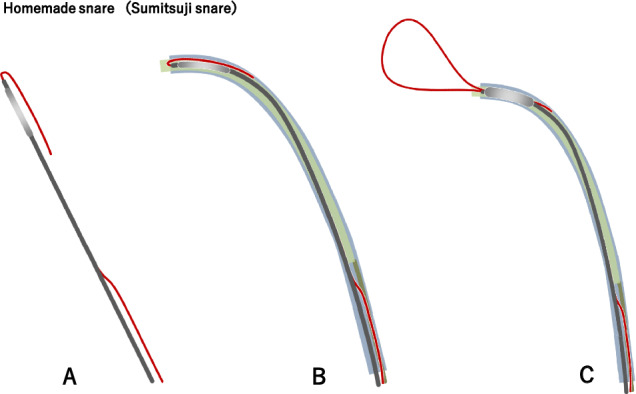
Homemade snare (Sumitsuji snare). Use GECs and balloons larger than 2 mm. **A** The normal-use wire is folded 20 mm from the tip of the monorail balloon as a reverse wire. The balloon diameter should be selected to ensure sufficient crimping of the guiding catheter during extension. **B** The tips of the folded wire and balloon are advanced close to the tip of the guiding extension catheter. **C**The reverse portion of the wire is fixed. After balloon dilation, a snare loop is created by pushing the wire forward through the balloon

When it is difficult to grip the proximal portion of the stent with a snare and the center portion of the stent is gripped, it is difficult to retrieve the stent into the guide catheter. In this situation, when the stent is pulled closer to the guide catheter, operators need to pull the system from the coronary artery ostium to the ascending aorta. Then, operators need to remove the guidewire and pull the snare harder to create a T-shaped stent at the edge of the guide catheter. The stent is folded in folio and retrieved into the guiding catheter. This technique can be used for guide catheters of 6 Fr or more, but some 6F guide catheters may have difficulty retrieving the stent. It should be noted that the stent can migrate into the head and neck vessels if the stent is not sufficiently grasped at the time of retrieval. If it is difficult to deliver the snare, it can be mounted on a small-diameter balloon to reach the stent site (Fig. 16). Simultaneous insertion of a small-diameter balloon and snare system is possible even with 6Fr guide catheter. In trans-radial PCI, retrieval should be switched to the femoral artery if the stent could not be retrieved into the guide catheter after successful grasping with a snare. Retrieval from the radial sheath with the exposed stent is not recommended due to the high risk of vascular injury. For the transition to retrieval from the femoral artery, the operator should insert a 0.025″ guidewire into the guide catheter, raise it to the origin of the right brachiocephalic or left subclavian arteries, and insert a 0.025″ guidewire into the descending aorta to guide the guide catheter and grasping the stent along it into the descending aorta. If this procedure is difficult, induction by grabbing and pulling a 0.025″ guidewire with snare from femoral should be considered. If a grasped stent is released during this procedure, there is a risk of migration into the arteries of the head and neck. Therefore, careful procedures must be warranted. The EN Snare should be used to retrieval via 8 Fr guide catheter from the femoral artery. If only the stent and snare guidewire can be grabbed by the EN Snare when retrieved from the femoral artery, the snare guidewire can be retrieved from the femoral artery together with the stent. If the microcatheters of the Goose Neck Snare are grabbed together, the hub of the microcatheter should be cut and retrieved from the femoral artery. If there is no EN Snare, it is useful to make a Sumitsuji snare (homemade snare; Fig. 17) and attempt to retrieve it [53].

Multi-guidewire approach: The operator adds two guidewires outside the stent. Both guidewires are advanced to another branch beyond the stent, and three guidewires are placed in one torquer and turned in the same direction. When the stent begins to rotate, it would be moved to the proximal portion while maintaining rotation. When retracting into the guide catheter, it is essential to confirm the coaxiality between the stent and guide catheter using fluoroscopy in multiple directions and to fine-tune the position of the guide catheter (height and direction). Operators should maintain rotation until the stent is entirely pulled into the guide catheter and then remove the entire system with the guide catheter, taking care not to inject the stent from the guide catheter. This method has several drawbacks including the entrapment of previously deployed stent and the possibility of losing the original guidewire. Additionally, rotation must be maintained when pulling the stent into the guide catheter, and the stent may be released from multiple guidewires in the ascending aorta during the pull-in phase. Therefore, this method is not recommended considering the low success rate and the risk of stent migration. If there is no obvious deformation in the dislodged stent and the guidewire is not inside the stent, the operator can attempt to insert the guidewire into the dislodged stent. If it is difficult to insert the guidewire into the stent, the guidewire should be advanced beside the dislodged and another stent can be inflated to fix the dislodged stent. If the site of dislodged stent is unacceptable to add another stent, attempts should be made to retrieve the dislodged stent. In this case, the guidewire is advanced beyond the dislodged stent, and the snare can be advanced through the guidewire to retrieve the dislodged stent using the method described above.

## Undeflatable balloon

Undeflatable balloon is a rare but serious complication in PCI. Undeflatable balloon occludes the coronary artery completely, which results in critical ischemia. Therefore, undeflatable balloon requires emergent bailout.

[Mechanism]

Causes include mechanical obstruction, kinking of the shaft, closure of the lumen that supplies fluid into balloon, and obstruction of the lumen by foreign objects or solids such as crystallized contrast media [54]. In particular, the balloon shaft is over-stretched or the inflation lumen is narrowed, when the balloon catheter is pulled hard before balloon is deflated or when the protective sheath is pulled out roughly. In addition, twisting of the balloon during the rewrapping process by turning or forcing the protective sheath may narrow the inflation lumen. This may result in a check-valve condition that would make deflation difficult.

[Management]

**Fig. 18:**
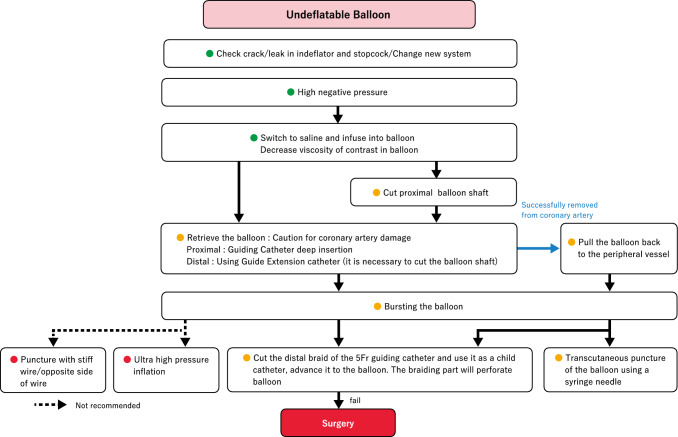
Flowchart for undeflatable balloon. The tip of the Heartrail II 5 Fr ST01 catheter (Terumo) is cut to expose the blade and inserted into the guiding catheter as a pediatric catheter. The catheter is placed close to the balloon, and its tip is pressed against the balloon to burst it. If the balloon is located distally, the catheter may be unable to reach it. Thus, the Y-connector should be replaced with a short hemostasis valve in advance. If the balloon cannot be ruptured by pressing the cut child’s catheter, the catheter is rotated slightly

A management flowchart is shown in Fig. 18. Leakage and damage in the indeflator and three-way stopcock should be evaluated. If damage is detected, the inflation system should be replaced with a new system. Subsequently, negative pressure is applied. To apply stronger negative pressure, it is considered to use two indeflators connected to each other. A 30 cc back-locking syringe is also considered. If a back-locking syringe is unavailable, a 10 cc syringe is inserted into the push-piece of the 30 cc syringe to maintain negative pressure. The contrast medium in the indeflator is changed to saline, and saline solution is slowly injected into the balloon. Decreasing the viscosity of the fluid by diluting it with saline may aid balloon deflation, because the negative pressure required to remove a less viscous fluid is smaller than that required to remove a higher-viscosity fluid. However, excessive negative pressure should be avoided, because excessive negative pressure may cause rupture in the balloon shaft. If the balloon shaft is cut at the most proximal end, the contrast agent may drain slowly and spontaneously. However, it is impossible to inflate the balloon after this procedure.

### Attempt to retrieve the entrapped balloon

If the trapped balloon is around the proximal site of coronary artery, manual traction can be tried after the guide catheter is gently inserted deep into the coronary artery to avoid damage to the coronary artery by the tip of guide catheter. If the trapped balloon is located at the distal site of coronary artery, manual traction can be tried after cutting the balloon hub and inserting the GEC via the balloon shaft into the coronary artery. Excessive traction may cause coronary artery injury and stretching or tearing of the balloon shafts. If the balloon is successfully removed from the coronary artery, it is important to pull the balloon into the peripheral artery. It is safer to continue the procedure in peripheral arteries than in coronary arteries.

### Bursting the entrapped balloon

It is an option to burst the balloon using a child catheter [55]. The tip of the child’s catheter is cut approximately 5 mm from the distal end. The purpose of cutting is not to sharpen the tip of the catheter, but to expose the blades in child catheters. The balloon burst is supposed to occur when exposed blades attach the tail of balloon. Therefore, the more exposed blades would result in greater success of this technique. The 5 Fr ST01 (Terumo, Tokyo, Japan) is strongly recommended as a child catheter for this technique because of braided blades. The child catheter is inserted into the mother catheter by covering the balloon shaft and guidewire while anchoring the system with entrapped balloon. If the balloon is located distally, the child catheter may not reach the balloon. The Y-connector should be replaced with a short-type hemostasis valve in advance. If the balloon could not be ruptured by pressing the cut child’s catheter, the catheter can be rotated slightly. GECs are often ineffective because they do not provide a sufficient push force or rotational manipulation. In addition, inserting a child catheter with a cut tip into the coronary artery increases the risk of vascular injury. If the balloon is positioned at the proximal site of coronary artery, the guide catheter can be inserted deeply. However, if the balloon is positioned at the distal site of coronary artery, the tip of the catheter cannot be protected by the guiding catheter, which requires more careful manipulation.

Another option is to perforate the balloon using the opposite side of the guidewire or stiff guidewires used in CTO PCI. Although there have been several successful case reports [54, 56–58], this method is not recommended because of its low success rate and the risk of vascular injury. Although stiff guidewires have good visibility under fluoroscopy, it often slips when the guidewire hits the occluded balloon. Therefore, stiff guidewires cannot puncture the occluded balloon. The opposite side (the tail) of the guidewire is not visible on fluoroscopy. Therefore, neither method is recommended because of the high risk of vascular injury. Another option is to apply a pressure beyond the rated burst pressure and to burst the occluded balloon. This method is not recommended because it may cause vascular injury or unexpected rupture of the shaft proximal to the occlusion rather than the balloon portion.

If the balloon can be pulled into peripheral arteries but is difficult to retrieve from the sheath, breaking the balloon with a 23 G needle has been reported [59]. Percutaneous puncture can easily be performed by palpating the balloon or using fluoroscopy. Although puncturing the balloon catheter alone may be sufficient to remove it from the sheath, aspirating the contrast medium from the balloon using a syringe at the time of puncture may be beneficial because it promotes deflation. Since these bursting balloon procedures carry the risk of vascular injury, covered stents should always be prepared in catheter rooms. If the above techniques are unsuccessful, it is important to request surgical removal immediately [60].

## Summary

Bailout methods for various complications described in this consensus document are based on taskforce members’ experiences, discussions, bench tests, and published literatures [3, 4, 32, 33, 48, 59, 61]. Among many bailout methods, it is difficult to recommend all these options, considering their effectiveness and reliability. Therefore, we do not recommend complex or unreliable strategies but recommended simple and reliable strategies, which are easy to understand and easy to perform even for inexperienced operators. In emergent situations, the contents of this document may not completely be applicable to real clinical practice, but can be a useful reference in real clinical practice. Furthermore, the recommended methods have the risk of unsuccessful bailout and subsequent complications. It is important for readers to understand that some recommended methods refer to unusual use or off-label use of devices.

PCI is the minimally invasive procedure with relatively low complication rate. However, once complications occur, operators must manage complications appropriately. Complication management requires preparation before PCI, early detection, emergent response, knowledge of bailout techniques, team-based communication, and multidisciplinary collaboration. This consensus document is expected to help operators to improve procedural outcomes in PCI with complications.

## References

[1] Yamaji K, Kohsaka S, Inohara T, Numasawa Y, Ando H, Wada H, Ishii H, Amano T, Miyata H, Ikari Y. Percutaneous coronary intervention during the COVID-19 pandemic in Japan: insights from the nationwide registration data. Lancet Reg Health West Pac. 2022;22: 100434. 10.1016/j.lanwpc.2022.100434. 35330940 10.1016/j.lanwpc.2022.100434 PMC8939342

[2] Nakamura M, Yaku H, Ako J, Arai H, Asai T, Chikamori T, Daida H, Doi K, Fukui T, Ito T, et al. JCS/JSCVS 2018 guideline on revascularization of stable coronary artery disease. Circ J. 2022;86:477–588. 10.1253/circj.CJ-20-1282. 35095031 10.1253/circj.CJ-20-1282

[3] Giannini F, Candilio L, Mitomo S, Ruparelia N, Chieffo A, Baldetti L, Ponticelli F, Latib A, Colombo A. A practical approach to the management of complications during percutaneous coronary intervention. JACC Cardiovasc Interv. 2018;11:1797–810. 10.1016/j.jcin.2018.05.052. 30236352 10.1016/j.jcin.2018.05.052

[4] Doll JA, Hira RS, Kearney KE, Kandzari DE, Riley RF, Marso SP, Grantham JA, Thompson CA, McCabe JM, Karmpaliotis D, et al. Management of percutaneous coronary intervention complications: algorithms from the 2018 and 2019 Seattle percutaneous coronary intervention complications conference. Circ Cardiovasc Interv. 2020;13: e008962. 10.1161/circinterventions.120.008962. 32527193 10.1161/CIRCINTERVENTIONS.120.008962

[5] Klaudel J, Glaza M, Klaudel B, Trenkner W, Pawłowski K, Szołkiewicz M. Catheter-induced coronary artery and aortic dissections. A study of the mechanisms, risk factors, and propagation causes. Cardiol J. 2022. 10.5603/CJ.a2022.0050. 35762078 10.5603/CJ.a2022.0050 PMC11229813

[6] Eshtehardi P, Adorjan P, Togni M, Tevaearai H, Vogel R, Seiler C, Meier B, Windecker S, Carrel T, Wenaweser P, Cook S. Iatrogenic left main coronary artery dissection: incidence, classification, management, and long-term follow-up. Am Heart J. 2010;159:1147–53. 10.1016/j.ahj.2010.03.012. 20569732 10.1016/j.ahj.2010.03.012

[7] Gómez-Moreno S, Sabaté M, Jiménez-Quevedo P, Vázquez P, Alfonso F, Angiolillo DJ, Hernández-Antolín R, Moreno R, Bañuelos C, Escaned J, Macaya C. Iatrogenic dissection of the ascending aorta following heart catheterisation: incidence, management and outcome. EuroIntervention. 2006;2:197–202. 19755261

[8] Núñez-Gil IJ, Bautista D, Cerrato E, Salinas P, Varbella F, Omedè P, Ugo F, Ielasi A, Giammaria M, Moreno R, et al. Incidence, management, and immediate- and long-term outcomes after iatrogenic aortic dissection during diagnostic or interventional coronary procedures. Circulation. 2015;131:2114–9. 10.1161/circulationaha.115.015334. 25888682 10.1161/CIRCULATIONAHA.115.015334

[9] Shaukat A, Tajti P, Sandoval Y, Stanberry L, Garberich R, Nicholas Burke M, Gössl M, Henry T, Mooney M, Sorajja P, et al. Incidence, predictors, management and outcomes of coronary perforations. Catheter Cardiovasc Interv. 2019;93:48–56. 10.1002/ccd.27706. 30312992 10.1002/ccd.27706

[10] Matsuura H, Mukai Y, Honda Y, Nishino S, Kang H, Kadooka K, Ogata K, Kimura T, Koiwaya H, Nishihira K, et al. Intra- and postprocedural management of coronary artery perforation during percutaneous coronary intervention. Circ Rep. 2022;4:517–25. 10.1253/circrep.CR-22-0092. 36408355 10.1253/circrep.CR-22-0092 PMC9638517

[11] Ellis SG, Ajluni S, Arnold AZ, Popma JJ, Bittl JA, Eigler NL, Cowley MJ, Raymond RE, Safian RD, Whitlow PL. Increased coronary perforation in the new device era. Incidence, classification, management, and outcome. Circulation. 1994;90:2725–30. 10.1161/01.cir.90.6.2725. 7994814 10.1161/01.cir.90.6.2725

[12] Al-Lamee R, Ielasi A, Latib A, Godino C, Ferraro M, Mussardo M, Arioli F, Carlino M, Montorfano M, Chieffo A, Colombo A. Incidence, predictors, management, immediate and long-term outcomes following grade III coronary perforation. JACC Cardiovasc Interv. 2011;4:87–95. 10.1016/j.jcin.2010.08.026. 21251634 10.1016/j.jcin.2010.08.026

[13] Chen H, Liu Y, Slipchenko MN, Zhao X, Cheng JX, Kassab GS. The layered structure of coronary adventitia under mechanical load. Biophys J. 2011;101:2555–62. 10.1016/j.bpj.2011.10.043. 22261042 10.1016/j.bpj.2011.10.043 PMC3297804

[14] Sugimoto T, Nomura T, Miyawaki D, Kato T, Keira N, Tatsumi T. Seesaw double GuideLiner(®) catheter technique for a successful bail-out procedure from blow-out type coronary perforation. Cardiovasc Interv Ther. 2017;32:396–400. 10.1007/s12928-016-0436-7. 27797010 10.1007/s12928-016-0436-7

[15] Goel PK. Delayed and repeated cardiac tamponade following microleak in RCA successfully treated with intra arterial sterile glue injection. Catheter Cardiovasc Interv. 2009;73:797–800. 10.1002/ccd.21924. 19301354 10.1002/ccd.21924

[16] Al Mawed M, Vlachojannis M, Pula A, Gielen S. Delayed coronary perforation four days after percutaneous coronary intervention with subsequent cardiac tamponade: a case report. Catheter Cardiovasc Interv. 2023;102:1061–5. 10.1002/ccd.30861. 37855161 10.1002/ccd.30861

[17] Fujimoto Y, Tonoike N, Kobayashi Y. Successful delivery of polytetrafluoroethylene-covered stent using rapid exchange guide extension catheter. Cardiovasc Interv Ther. 2017;32:142–5. 10.1007/s12928-016-0378-0. 26815132 10.1007/s12928-016-0378-0

[18] Danek BA, Karatasakis A, Brilakis ES. Consequences and treatment of guidewire entrapment and fracture during percutaneous coronary intervention. Cardiovasc Revasc Med. 2016;17:129–33. 10.1016/j.carrev.2015.12.005. 26826011 10.1016/j.carrev.2015.12.005

[19] Hartzler GO, Rutherford BD, McConahay DR. Retained percutaneous transluminal coronary angioplasty equipment components and their management. Am J Cardiol. 1987;60:1260–4. 10.1016/0002-9149(87)90604-7. 2961239 10.1016/0002-9149(87)90604-7

[20] Gasparini GL, Sanz-Sanchez J, Regazzoli D, Boccuzzi G, Oreglia JA, Gagnor A, Mazzarotto P, Belli G, Garbo R. Device entrapment during percutaneous coronary intervention of chronic total occlusions: incidence and management strategies. EuroIntervention. 2021;17:212–9. 10.4244/eij-d-20-00781. 32894229 10.4244/EIJ-D-20-00781 PMC9724869

[21] Lotan C, Hasin Y, Stone D, Meyers S, Applebaum A, Gotsman MS. Guide wire entrapment during PTCA: a potentially dangerous complication. Catheter Cardiovasc Diagn. 1987;13:309–12. 10.1002/ccd.1810130505. 10.1002/ccd.1810130505 2959368

[22] Ghosh PK, Alber G, Schistek R, Unger F. Rupture of guide wire during percutaneous transluminal coronary angioplasty. Mechanics and management. J Thorac Cardiovasc Surg. 1989;97:467–9. 2521914

[23] Karabulut A, Daglar E, Cakmak M. Entrapment of hydrophilic coated coronary guidewire tips: which form of management is best? Cardiol J. 2010;17:104–8. 20104468

[24] Iturbe JM, Abdel-Karim AR, Papayannis A, Mahmood A, Rangan BV, Banerjee S, Brilakis ES. Frequency, treatment, and consequences of device loss and entrapment in contemporary percutaneous coronary interventions. J Invas Cardiol. 2012;24:215–21. 22562915

[25] Numasawa Y, Sakakura K, Yamamoto K, Yamamoto S, Taniguchi Y, Fujita H, Momomura SI. A novel side branch protection technique in coronary stent implantation: Jailed Corsair technique. Cardiovasc Revasc Med. 2017;18:295–8. 10.1016/j.carrev.2017.01.009. 28119044 10.1016/j.carrev.2017.01.009

[26] Shishido K, Moriyama N, Hayashi T, Yokota S, Miyashita H, Mashimo Y, Yokoyama H, Nishimoto T, Ochiai T, Tobita K, et al. The efficacy of modified jailed balloon technique for true bifurcation lesions. Catheter Cardiovasc Interv. 2020;96:20–8. 10.1002/ccd.28812. 32096918 10.1002/ccd.28812

[27] Kilic H, Akdemir R, Bicer A. Rupture of guide wire during percutaneous transluminal coronary angioplasty, a case report. Int J Cardiol. 2008;128:e113-114. 10.1016/j.ijcard.2007.05.088. 17692955 10.1016/j.ijcard.2007.05.088

[28] Devidutta S, Lim ST. Twisting wire technique: an effective method to retrieve fractured guide wire fragments from coronary arteries. Cardiovasc Revasc Med. 2016;17:282–6. 10.1016/j.carrev.2016.01.013. 27106743 10.1016/j.carrev.2016.01.013

[29] Leibundgut G, Achim A, Krivoshei L. Safe and predictable transcatheter removal of broken coronary guidewires: the “knuckle-twister” technique: a case series report. Eur Heart J Case Rep. 2023;7:ytad11. 10.1093/ehjcr/ytad311. 10.1093/ehjcr/ytad311 PMC10394303 37539349

[30] Burns AT, Gutman J, Whitbourn R. Side-branch wire entrapment during bifurcation PCI: avoidance and management. Catheter Cardiovasc Interv. 2010;75:351–3. 10.1002/ccd.22269. 19806638 10.1002/ccd.22269

[31] Tanaka K, Okamura A, Iwamoto M, Watanabe S, Nagai H, Sumiyoshi A, Suzuki S, Tanaka H, Koyama Y, Iwakura K, Fujii K. Wire cutting method using rotational atherectomy for stretched spring wire during coronary intervention. JACC Case Rep. 2021;3:1842–8. 10.1016/j.jaccas.2021.09.018. 34917965 10.1016/j.jaccas.2021.09.018 PMC8642729

[32] Hiraya D, Sato A, Hoshi T, Sakai S, Watabe H, Ieda M. Incidence, retrieval methods, and outcomes of intravascular ultrasound catheter stuck within an implanted stent: systematic literature review. J Cardiol. 2020;75:164–70. 10.1016/j.jjcc.2019.07.005. 31416780 10.1016/j.jjcc.2019.07.005

[33] Sakakura K, Ito Y, Shibata Y, Okamura A, Kashima Y, Nakamura S, Hamazaki Y, Ako J, Yokoi H, Kobayashi Y, Ikari Y. Clinical expert consensus document on rotational atherectomy from the Japanese association of cardiovascular intervention and therapeutics. Cardiovasc Interv Ther. 2021;36:1–18. 10.1007/s12928-020-00715-w. 33079355 10.1007/s12928-020-00715-w PMC7829233

[34] Sakakura K, Ito Y, Shibata Y, Okamura A, Kashima Y, Nakamura S, Hamazaki Y, Ako J, Yokoi H, Kobayashi Y, Ikari Y. Clinical expert consensus document on rotational atherectomy from the Japanese association of cardiovascular intervention and therapeutics: update 2023. Cardiovasc Interv Ther. 2023;38:141–62. 10.1007/s12928-022-00906-7. 36642762 10.1007/s12928-022-00906-7 PMC10020250

[35] Sakakura K, Ako J, Wada H, Naito R, Funayama H, Arao K, Kubo N, Momomura S. Comparison of frequency of complications with on-label versus off-label use of rotational atherectomy. Am J Cardiol. 2012;110:498–501. 10.1016/j.amjcard.2012.04.021. 22579342 10.1016/j.amjcard.2012.04.021

[36] Kaneda H, Saito S, Hosokawa G, Tanaka S, Hiroe Y. Trapped rotablator: kokesi phenomenon. Catheter Cardiovasc Interv. 2000;49:82–4. 10.1002/(sici)1522-726x(200001)49:1<82::aid-ccd18>3.0.co;2-x**(discussion 85)**. 10.1002/(sici)1522-726x(200001)49:1<82::aid-ccd18>3.0.co;2-x 10627374

[37] Sakakura K, Ako J, Momomura S. Successful removal of an entrapped rotablation burr by extracting drive shaft sheath followed by balloon dilatation. Catheter Cardiovasc Interv. 2011;78:567–70. 10.1002/ccd.22957. 21780279 10.1002/ccd.22957

[38] Grise MA, Yeager MJ, Teirstein PS. A case of an entrapped rotational atherectomy burr. Catheter Cardiovasc Interv. 2002;57:31–3. 10.1002/ccd.10263. 12203923 10.1002/ccd.10263

[39] Sakakura K, Taniguchi Y, Tsukui T, Yamamoto K, Momomura SI, Fujita H. Successful removal of an entrapped rotational atherectomy burr using a soft guide extension catheter. JACC Cardiovasc Interv. 2017;10:e227–9. 10.1016/j.jcin.2017.09.036. 29153503 10.1016/j.jcin.2017.09.036

[40] Hyogo M, Inoue N, Nakamura R, Tokura T, Matsuo A, Inoue K, Tanaka T, Fujita H. Usefulness of conquest guidewire for retrieval of an entrapped rotablator burr. Catheter Cardiovasc Interv. 2004;63:469–72. 10.1002/ccd.20232. 15558759 10.1002/ccd.20232

[41] Tanaka Y, Saito S. Successful retrieval of a firmly stuck rotablator burr by using a modified STAR technique. Catheter Cardiovasc Interv. 2016;87:749–56. 10.1002/ccd.26342. 26651133 10.1002/ccd.26342

[42] Kimura M, Shiraishi J, Kohno Y. Successful retrieval of an entrapped rotablator burr using 5 Fr guiding catheter. Catheter Cardiovasc Interv. 2011;78:558–64. 10.1002/ccd.22995. 21547995 10.1002/ccd.22995

[43] Kanazawa T, Kadota K, Mitsudo K. Successful rescue of stuck rotablator burr entrapment using a Kiwami straight catheter. Catheter Cardiovasc Interv. 2015;86:942–5. 10.1002/ccd.25903. 25712486 10.1002/ccd.25903

[44] Tehrani S, Achan V, Rathore S. Percutaneous retrieval of an entrapped rotational atherectomy burr using novel technique of controlled traction and counter traction. Cardiovasc Revasc Med. 2021;28s:132–5. 10.1016/j.carrev.2020.11.002. 33191146 10.1016/j.carrev.2020.11.002

[45] Prasan AM, Patel M, Pitney MR, Jepson NS. Disassembly of a rotablator: getting out of a trap. Catheter Cardiovasc Interv. 2003;59:463–5. 10.1002/ccd.10611. 12891607 10.1002/ccd.10611

[46] Assad-Kottner C, Hakeem A, Uretsky BF. Modified dual guide catheter (“ping-pong”) technique to treat left internal mammary artery graft perforation. Catheter Cardiovasc Interv. 2015;86:E28-31. 10.1002/ccd.25598. 25044448 10.1002/ccd.25598

[47] Gambhir DS, Batra R, Singh S, Kaul UA, Arora R. Burr entrapment resulting in perforation of right coronary artery: an unreported complication of rotational atherectomy. Indian Heart J. 1999;51:307–9. 10624071

[48] Brilakis ES, Best PJ, Elesber AA, Barsness GW, Lennon RJ, Holmes DR Jr, Rihal CS, Garratt KN. Incidence, retrieval methods, and outcomes of stent loss during percutaneous coronary intervention: a large single-center experience. Catheter Cardiovasc Interv. 2005;66:333–40. 10.1002/ccd.20449. 16142808 10.1002/ccd.20449

[49] Alomar ME, Michael TT, Patel VG, Altomare CG, Rangan BV, Cipher D, Banerjee S, Brilakis ES. Stent loss and retrieval during percutaneous coronary interventions: a systematic review and meta-analysis. J Invas Cardiol. 2013;25:637–41. 24296383

[50] Bolte J, Neumann U, Pfafferott C, Vogt A, Engel HJ, Mehmel HC, von Olshausen KE. Incidence, management, and outcome of stent loss during intracoronary stenting. Am J Cardiol. 2001;88:565–7. 10.1016/s0002-9149(01)01742-8. 11524072 10.1016/s0002-9149(01)01742-8

[51] Kühn AL, Singh J, Puri AS. Migrated coronary stent into the left internal carotid artery: a rescue technique. BMJ Case Rep. 2023. 10.1136/bcr-2023-257501. 37940198 10.1136/bcr-2023-257501 PMC10632800

[52] Ogawa T, Inoue Y, Aizawa T, Morimoto S, Ogawa K, Nagoshi T, Minai K, Kawai M, Yoshimura M. Investigation of the small-balloon technique as a method for retrieving dislodged stents. Cardiovasc Interv Ther. 2023;38:309–15. 10.1007/s12928-023-00917-y. 36800064 10.1007/s12928-023-00917-y

[53] Yokoi K, Sumitsuji S, Kaneda H, Siegrist PT, Okayama K, Ide S, Mizote I, Kumada M, Kuroda T, Tachibana K, et al. A novel homemade snare, safe, economical and size-adjustable. EuroIntervention. 2015;10:1307–10. 10.4244/eijv10i11a220. 24642569 10.4244/EIJV10I11A220

[54] Gilchrist IC. Troubleshooting and treating the balloon that fails to deflate. Catheter Cardiovasc Interv. 2011;77:62. 10.1002/ccd.22925. 21181971 10.1002/ccd.22925

[55] Takama T, Ito Y, Ishimori H, Tsukahara R, Muramatsu T. Failure of a balloon to deflate during post dilatation in a coronary artery. Cardiovasc Interv Ther. 2015;30:57–60. 10.1007/s12928-014-0249-5. 24532231 10.1007/s12928-014-0249-5

[56] Bostan M, Satiroğlu O, Erdoğan T, Durakoğlugil ME, Uğurlu Y. A rare complication: undeflatable balloon of the stent. Interv Med Appl Sci. 2013;5:43–5. 10.1556/imas.5.2013.1.9. 24265889 10.1556/IMAS.5.2013.1.9 PMC3831790

[57] Trivedi R. Double jeopardy: failure to deflate stent balloon in rescue angioplasty. Interv Med Appl Sci. 2019;11:128–30. 10.1556/1646.11.2019.16. 32148919 10.1556/1646.11.2019.16 PMC7044536

[58] Girish MP, Gupta MD, Tyagi S. Entrapped coronary angioplasty stent balloon due to nondeflation: percutaneous retrieval by a simple technique. Catheter Cardiovasc Interv. 2011;77:58–61. 10.1002/ccd.22617. 20506291 10.1002/ccd.22617

[59] Leibundgut G, Degen C, Riede F. Transcutaneous puncture of an undeflatable coronary angioplasty balloon catheter. Case Rep Cardiol. 2018;2018:6252809. 10.1155/2018/6252809. 30250754 10.1155/2018/6252809 PMC6140099

[60] Chang TM, Pellegrini D, Ostrovsky A, Marrangoni AG. Surgical management of entrapped percutaneous transluminal coronary angioplasty hardware. Tex Heart Inst J. 2002;29:329–32. 12484620 PMC140298

[61] Al-Moghairi AM, Al-Amri HS. Management of retained intervention guide-wire: a literature review. Curr Cardiol Rev. 2013;9:260–6. 10.2174/1573403x11309030010. 23116055 10.2174/1573403X11309030010 PMC3780351

